# Current Perspectives on Primary Immunodeficiency Diseases

**DOI:** 10.1080/17402520600800705

**Published:** 2006

**Authors:** Arvind Kumar, Suzanne S. Teuber, M. Eric Gershwin

**Affiliations:** Division of Rheumatology Allergy and Clinical Immunology Department of Internal Medicine University of California at Davis School of Medicine Davis CA USA

## Abstract

Since the original description of X-linked agammaglobulinemia in 1952, the number of independent primary immunodeficiency diseases (PIDs) has expanded to more than 100 entities. By definition, a PID is a genetically determined disorder resulting in enhanced susceptibility to infectious disease. Despite the heritable nature of these diseases, some PIDs are clinically manifested only after prerequisite environmental exposures but they often have associated malignant, allergic, or autoimmune manifestations. PIDs must be distinguished from secondary or acquired immunodeficiencies, which are far more common. In this review, we will place these immunodeficiencies in the context of both clinical and laboratory presentations as well as highlight the known genetic basis.


*Clinical & Developmental Immunology*, June–December 2006; 13(2–4): 223–259

Taylor & Francis

Taylor & Francis Group

### Current perspectives on primary immunodeficiency diseases

ARVIND KUMAR, SUZANNE S. TEUBER, & M. ERIC GERSHWIN

*Division of Rheumatology, Allergy and Clinical Immunology, Department of Internal Medicine, University of California at Davis School of Medicine, Davis, CA, USA*

**Abstract**

Since the original description of X-linked agammaglobulinemia in 1952, the number of independent primary immunodeficiency diseases (PIDs) has expanded to more than 100 entities. By definition, a PID is a genetically determined disorder resulting in enhanced susceptibility to infectious disease. Despite the heritable nature of these diseases, some PIDs are clinically manifested only after prerequisite environmental exposures but they often have associated malignant, allergic, or autoimmune manifestations. PIDs must be distinguished from secondary or acquired immunodeficiencies, which are far more common. In this review, we will place these immunodeficiencies in the context of both clinical and laboratory presentations as well as highlight the known genetic basis.

**Keywords:** *Primary immunodeficiency disease, primary immunodeficiency, immunodeficiencies, autoimmune*

### Introduction

Acquired immunodeficiencies may be due to malnutrition, immunosuppressive or radiation therapies, infections (human immunodeficiency virus, severe sepsis), malignancies, metabolic disease (diabetes mellitus, uremia, liver disease), loss of leukocytes or immunoglobulins (Igs) via the gastrointestinal tract, kidneys, or burned skin, collagen vascular disease such as systemic lupus erythematosis, splenectomy, and bone marrow transplant (BMT) (Tangsinmankong et al. 2001). The importance of gaining a fuller understanding of the PIDs lies in the difficulties of diagnosis, their potentially severe clinical manifestations as well as the fact that their study provides insight into basic immunologic mechanisms in health and disease. With this in mind, the focus of this article will be to describe some of the most representative, clinically significant PIDs.

### Classification of PIDs

In 1970, a committee of the World Health Organization (WHO) classified the then fourteen known PIDs into a uniform nomenclature (Chapel et al. 2003). The International Union of Immunological Societies (IUIS) has subsequently convened an international committee of experts every two to three years to revise this classification based on new PIDs and further understanding of the molecular basis. A recent IUIS committee met in 2003 in Sintra, Portugal with its findings published in 2004 in the *Journal of Allergy and Clinical Immunology* (Chapel et al. 2003). The last IUIS meeting took place in June 2005 in Budapest, Hungary, with their findings published in the April 2006 issue of the *JACI*. The next WHO/IUIS Expert Meeting will be in May 2007.

PIDs may involve one or multiple components of the immune system, i.e. B cells, T cells, natural killer (NK) cells, phagocytes, complement and/or the immune mechanisms that link these components, such as the major histocompatibility complex (MHC) I and II. Although some authors group PIDs by known vs. unknown associated molecular defects, the generic classification divides PIDs into broad deficiencies: humoral, cell-mediated, combined humoral and cell-mediated, phagocyte, complement pathway, and other well-characterized immunodeficiency syndromes of

Correspondence: M. E. Gershwin, Division of Rheumatology, Allergy and Clinical Immunology, University of California at Davis School of Medicine, 451 E. Health Sciences Drive, Suite 6510, Davis, CA 95616, USA. Tel: 1 530 752 2884. Fax: 1 530 752 4669. E-mail: megershwin@ucdavis.edu

ISSN 1740-2522 print/ISSN 1740-2530 online © 2006 Taylor & Francis
DOI: 10.1080/17402520600800705

uncertain molecular mechanism (Chapel et al. 2003). Some further divide the combined immunodeficiencies into severe combined immunodeficiency disease (SCIDs) and combined immunodeficiency disease (CID), with SCID implying a more significant cellular immune deficiency than CID. However, given the variability in presentation and severity in these disorders, these groups may overlap and are sometimes not subdivided (Notarangelo et al. 2004). SCIDs are also sometimes described as $T^{-}B^{+}$ to indicate T cell deficiency with relatively preserved B cell numbers or $T^{-}B^{-}$ to signify the absence of both T and B cells (Notarangelo et al. 2004).

### Genetic basis of PID

Most PIDs are secondary to an abnormality of a single gene and most are autosomal recessive (Buckley 2003a, Notarangelo et al. 2004). A few PIDs, including one of the most well known of such disorders, Bruton's agammaglobulinemia, are X-linked recessive. Advances in genetics and molecular biology techniques over the last two decades have allowed genetic identification and often the abnormal gene has been cloned, sequenced, and the product identified. Uncovering the pathophysiologic basis of certain PIDs in this fashion creates a foundation from which targeted therapy may be possible. The tables that follow identify the known genes and gene products that are affected in the various PIDs.

### Frequency of PIDs

Clearly, PIDs are uncommon. Estimates of incidence vary from less than 1 in 2 million live births for extremely rare conditions to as many as 1 in 333 for Ig A deficiency, the most frequently diagnosed PID (Cunningham-Rundles 2001, Vihinen 2004). However, as a group, PIDs may be as common as pediatric leukemia and lymphoma and four times as common as cystic fibrosis (Tangsinmankong et al. 2001). Humoral PIDs are the most common, representing more than half of cases, while cellular, or combined, or phagocyte disorders account for about 10–20% of cases (Matamoros Flori et al. 1997, Javier et al. 2000, Stray-Pedersen et al. 2000, Tangsinmankong et al. 2001). Complement pathway defects account for only 1–3% of cases (Matamoros Flori et al. 1997, Javier et al. 2000, Stray-Pedersen et al. 2000, Tangsinmankong et al. 2001).

### Commonalities and general rules

As alluded to above, many PIDs classically present during early life with recurrent infection, severe infection, difficult to control infection, or infection from opportunistic pathogens. In today's setting of frequent broad-spectrum antibiotic use, such classic presentations are often altered. Clinicians may not initially find such obvious susceptibility to infections and may face patients who present with autoimmune or allergic complaints (Buckley 2003a). This makes diagnosis all the more difficult, and necessitates a high index of suspicion for PIDs. PID is most often diagnosed in the pediatric age group, with more than 80% of cases diagnosed before age 20, but can present in adults (Lindegren et al. 2004, Riminton and Limaye 2004). There is a male predominance in children, but slight female predominance in those diagnosed as adults (Buckley 2003a).

Complications and pathogen susceptibility patterns vary according to the immune deficit. For example, B cell, phagocyte, or complement abnormalities often result in recurrent encapsulated bacterial infections, while T cell abnormalities lead to opportunistic infections from viral and fungal organisms and failure to thrive. Combined PIDs typically include infections from pathogens of either or both groups. In a recent update on PID, Bonilla and Geha have summarized patterns of pathogen susceptibility for various PIDs (Figure 1).

### Humoral PIDs

PIDs that result in humoral, or antibody, deficiency are the most frequently encountered congenital immune deficiency. Humoral PIDs include X-linked agammaglobulinemia as well as several autosomal recessive disorders. Due to the delay in fetal production of antibody and the gradual loss of maternally derived IgG over the first six to twelve months of life, humoral PIDs often have a delayed presentation until six to twelve months of age (Bonilla and Geha 2003). Typical infectious problems include respiratory disease from encapsulated bacteria. Nonrespiratory infection and sepsis from these pathogens also occurs. Enteroviral gastrointestinal or systemic disease is also typical of humoral PID (Bonilla and Geha 2003; Figure 1). Appropriate use of antibiotics and regular intravenous Ig infusions are the foundation of therapy of most humoral PIDs. IVIG is contraindicated in certain diseases such as selective IgA deficiency and not indicated in others, such as most cases of IgG subclass deficiency. Table I lists the humoral PIDs, the genetic and molecular defects (if known) thought to be causative, and other immuno-clinical features of each disease. The most significant individual disorders are further described in the text below.

### Humoral PIDs

### *X-linked agammaglobulinemia (Bruton's agammaglobulinemia, XLA, Bruton's disease)*

XLA is the most common of the agammaglobulinemias, representing 80–90% of cases (Bonilla and

| Organism | Antibody deficiency | Cellular deficiency | Combined deficiency | Phagocyte defects | Complement deficiency |
|---|---|---|---|---|---|
| | | | | | |
| Viruses | Enteroviruses | Herpes virus | All | No | No |
| Bacteria | *Streptococcus pneumoniae, Haemophilus influenza, Staphylococcus aureus, Pseudomonas aeruginosa, Campylobacter fetus, Neisseria meningitides, Mycoplasma hominis, Ureaplasma ureolyticum* | *Salmonella typhi* | As for antibody deficiency, also: *Listeria monocytogenes, Salmonella typhi*, enteric flora | *S. aureus*, enteric flora, *P. aeruginosa, S. typhi, N.asteroids* | As for antibody deficiency, especially, *Neisseria meningitides* |
| Mycobacteria | No | All, including BCG | All, including BCG | All, including BCG | No |
| Fungi | No | *Candida albicans, Histoplasma capsulatum, Aspergillus fumigatus, Coccidioides immitis* | *Candida albicans, Cryptococcus neoformans, Pneumocystis carinii* | *Aspergillus fumigatus, Candida albicans, Pneumocystis carinii* | No |
| Protozoa | *Giardia lamblia* | | *Toxoplasma ondgii* | | No |

Adapted from Bonilla and Geha. J Allergy Clin Immunol. 2006; 117:S435-41 (Bonilla and Geha 2006)

Figure 1. Infectious organisms associated with major categories of immune deficiency.

Geha 2003) and the clinical manifestations are recurrent infections due to encapsulated bacteria including *Streptococcus pneumonia*, *Staphylococcus aureus*, *Haemophilus influenzae*, *Nesseria meningiditis* but also *Mycoplasma* and *Pseudomonas* species (Buckley 2003a). Although there is a delay in onset of most infections during the first few months of life due to maternal antibodies, these patients may have mucous membrane disease, i.e. conjunctivitis or otitis. This occurs since there is a lack of secretory IgA (Buckley 2003a). Once passive immunity from maternal IgG wanes, the almost complete absence of Igs of any isotype allows recurrent infection, especially mucous membrane disease (pneumonia, otitis, gastroenteritis, urinary tract infection), systemic infection (meningitis, sepsis), osteomyelitis, septic arthritis, cellulitis, and skin abscesses (Timmers et al. 1991, Bonilla and Geha 2003). Enteroviruses such as Poliovirus from live virus-vaccines can lead to viremia and subsequent CNS disease, paralysis, and death (Mellor 1981). Hepatitis is also a possible viral complication (Buckley 2003a). Despite these frequent infections, patients with XLA typically do not have failure to thrive unless they develop bronchiectasis or persistent enteroviral disease (Buckley 2003a). As with most humoral PIDs, these patients do not usually get fungal, mycobacterial, or non-enterovirus viral infections. On physical exam, patients have small or absent lymphoid tissue, including tonsils, adenoids, and peripheral lymph nodes due to abnormal B cell development (Buckley 2003a). A few patients with XLA have had associated growth hormone deficiency (Buzi et al. 1994).

Although Bruton recognized this disease in 1952, the molecular basis for XLA was not identified until 1993 (Tsukada et al. 1993, Vetrie et al. 1993) as a cytoplasmic tyrosine kinase known as Bruton tyrosine kinase (Btk). Btk is found in many cells of hematopoiesis, and large amounts of the kinase are produced normally in all B cells and B cell precursors, but not in T cells (de Weers et al. 1993). This kinase is essential for intracellular signal transduction that must occur in bone marrow pre-B cells in order for maturation to B cells and antibody-producing plasma cells (Tsukada et al. 1993, Vetrie et al. 1993). Hundreds of mutations in the human Btk gene have been identified (Vihinen et al. 2001) and all patients with XLA have had low or undetectable levels of Btk messenger ribonucleic acid and kinase activity (Buckley 2003a).

Immunologically, XLA presents with almost non-existent concentrations of Igs of all isotypes. These patients will not demonstrate isohemagglutinins, nor appropriate antibody production after immunization with protein or polysaccharide vaccines (Buckley 2003a). Bone marrow analysis may demonstrate some pre-B cells, but flow cytometry will reveal few to no circulating B cells or plasma cells (Buckley 2003a). T cell and NK cell numbers may be increased in the circulation, and they function normally (Buckley 2003a). CD4/CD8 ratios, thymus, and T-cell zones of lymphoid tissues are also normal in XLA (Buckley 2003a). Granulocyte function is normal if patients are given IgG, but a few patients with XLA develop transient neutropenia without cause or at the start of a severe infection (Cham et al. 2002, Buckley 2003a). This may be related to the fact that Btk is also found in myeloid cell lineages (Cham et al. 2002).

The mainstay of treatment for XLA, and most humoral PIDs, is regular infusion of intravenous Ig (IVIG) (Aghamohammadi et al. 2004). If IVIG is started early, patients have a good prognosis. However, some patients develop persistent enteroviral

Table I. Humoral PIDs.

| Disorder | Presumed pathogenetic mechanism: Abnormal gene | Abnormal genetic locus | Abnormal gene product | Classic/associated features | T cell # (blood) | B cells # (blood) | Serum Ig | Inheritance |
|---|---|---|---|---|---|---|---|---|
| Agammaglobulinemias | | | | | | | | |
| X-linked agammaglobulinemia (Bruton's agammaglobulinemia, XLA) | *BTK* | Xq22 | Btk (Bruton tyrosine kinase) | Severe bacterial infection; enteroviral infection; possible rheumatoid arthritis/ malignancy | N or ↑ | ↓↓ | ↓↓ ALL | XL |
| IgM heavy chain defect (μ defect) | *IGHM* | 14q32.3 | μ (IgM heavy chain) | Same as XLA | Same as XLA | Same as XLA | Same as XLA | AR |
| Ig-α defect (CD79a defect) | *CD79A* | 19p13.2 | Ig-α | Same as XLA | Same as XLA | Same as XLA | Same as XLA | AR |
| Surrogate light chain defect (λ5 deficiency, CD179b deficiency) | *CD179B* | 22q11.2 | Surrogate light chain | Same as XLA | Same as XLA | Same as XLA | Same as XLA | AR |
| B cell-linker protein (BLNK) defect | *BLNK* | 10q23.2–q23.33 | BLNK | Same as XLA | Same as XLA | Same as XLA | Same as XLA | AR |
| Leucine-rich repeat-containing 8 gene defect (LRRC8 defect) | *LRRC8* | 9q33.2 | LRRC8 | Same as XLA | Same as XLA | Same as XLA | Same as XLA | AR |
| Hyper IgM syndromes, autosomal recessive type | | | | | | | | |
| Activation-induced cytidine deaminase (AICD) defect | *AICD* | 12p13 | AID | Severe bacterial infection; enlarged lymph nodes and germinal centers | N | N | High IgM; others low | AR |
| Uracil nucleoside glycosylase (UNG) defect | *UNG* | 17q11.2 | UNG | Severe bacterial infection; enlarged lymph nodes and germinal centers | N | N | High IgM; others low | AR |
| Others | | | | | | | | |
| Immunodeficiency, centromeric instability, facial anomalies (ICF)-syndrome | *DNMT3B* | 20q11.2 | DNA methyltransferase 3B | Recurrent respiratory bacterial infection in 2/3, abnormal facies in 2/3, pathognomonic centromere anomalies of chromosomes 1,9, or 16 | N | N | Variably ↓ | AR |
| κ light-chain deficiency | *IGKC* | 2p12 | κ light chain | Often asymptomatic | N | N or ↓ κ-bearing B cells | Ig(κ) ↓; Ab response Nl or ↓ | AR |
| Ig heavy chain gene deletions | – | 14q32 | – | Often asymptomatic | N | N or ↓ | IgG1, IgG2, or IgG4 absent & some with absent IgE and IgA1 or IgA2 | AR |
| CVID (subset with associated molecular defect) | | | | | | | | |
| Inducible T Cell costimulator (ICOS) defect | *ICOS* | 2q33 | ICOS | Recurrent bacterial infection, autoimmunity, splenomegaly | N | ↓ | ↓ All | AR |
| Transmembrane activator and calcium modulator and cyclophilin ligand interactor (TACI) Defect | *TNFRSF13B* | 17p11.2 | TACI | Recurrent bacterial infections, autoimmunity, hepatosplenomegaly, malignancy | N | ↓ | ↓ All | AR |

Table I – *continued*

| | Presumed pathogenetic mechanism | | | | | | | |
|---|---|---|---|---|---|---|---|---|
| Disorder | Abnormal gene | Abnormal genetic locus | Abnormal gene product | Classic/associated features | T cell # (blood) | B cells # (blood) | Serum Ig | Inheritance |
| Humoral PIDs with unknown molecular basis | | | | | | | | |
| CVID of unknown etiology | ? | ? | ? | Recurrent bacterial infections, autoimmunity, hepatosplenomegaly, collagen vascular disease, malignancy | N usually | $\downarrow$ or N | $\downarrow$ IgG and usually IgA $\pm$ IgM | Variable; Unknown |
| Selective IgA deficiency (IGDA) | *IGAD1* | 6p21.3 | ? | Often asymptomatic, possible bacterial infections, autoimmunity, collagen vascular disease, malignancy, atopy | N | N or $\downarrow$ sIga$^{+}$ cells | $\downarrow$ IgA1 and IgA2 | ? |
| Specific antibody deficiency with normal immunoglobulins (SADNI) | ? | ? | ? | Cannot make antibodies against specific antigens | N | N | N | ? |
| IgG subclass deficiency (IGGSD) | ? | ? | ? | Often asymptomatic | N | N or immature | $\downarrow$ in one or more IgG subtypes | ? |
| Transient Hypogammaglobulinemia of Infancy (THI) | ? | ? | ? | Usually mild respiratory infections | N | N | $\downarrow$ IgA and IgG | ? |

Data abstracted from Anonymous 2000, Buckley 2003a, Chapel et al. 2003, Notarangelo et al. 2004, Salzer et al. 2005, Vihinen 2004, Bonilla and Geha 2006. Abbreviations: AR, Autosomal recessive; XL, X-linked; SCID, severe combined immunodeficiency; $\downarrow$, decreased; $\downarrow\downarrow$, profoundly decreased; $\uparrow$, increased; N, normal; for Serum Ig Column, “All” refers to all isotypes.

infections, poliomyelitis, rheumatoid arthritis-like disease, or malignancies of the lymphoreticular or other systems (Hermaszewski et al. 1993, Lavilla et al. 1993, Lee et al. 1993, Sany et al. 1993, Filipovich et al. 1994, Buckley 2003a). These patients have a much poorer prognosis. The incidence of lymphoreticular malignancy in XLA patients is as high as 6% (Buckley 2003a). In addition, a significant number of patients without these complications may develop persistent enteroviral infection or severe sinopulmonary disease despite IVIG (Buckley 2003a). Many such patients are managed with prophylactic and long-term antibiotics (Table I).

*Autosomal recessive agammaglobulinemias: **μ** Deficiency (IgM heavy chain deficiency); B cell linker protein deficiency (BLNK defect); Ig-**α** deficiency (CD79a deficiency); surrogate light chain deficiency (**λ**5 deficiency, CD179b deficiency); and leucine-rich repeat-containing 8 gene defect (LRRC8 defect)*

The five defects listed above are autosomal recessive and patients have agammaglobulinemia or significant hypogammaglobulinemia (Yel et al. 1996, Minegishi et al. 1998, Minegishi et al. 1999, Wang et al. 2002, Sawada et al. 2003). They all present with clinical and immunologic phenotypes similar to XLA (Bonilla and Geha 2003) but are much more uncommon. Mutations in the μ heavy chain gene have been reported in approximately twelve patients, while there have only been single reports of the other defects in humans (Bonilla and Geha 2003). LRRC8 defect was associated with abnormal facies in the affected girl (Sawada et al. 2003). IgM heavy chain deficiency, BLNK deficiency, Ig-α deficiency, and surrogate light chain deficiency all cause arrest of B cell development at the pre-B cell stage in the bone marrow (Buckley 2003a). This is because development of the pre-B cell is dependent on signal transduction through the pre-B cell receptor (Buckley 2003a). The pre-B cell receptor consists of IgM heavy chain, surrogate light chain (in a heterodimer with VpreB), and Ig-α (in a heterodimer with Ig-β). Defects in these components prevent expression of the pre-B cell receptor on the cell surface, leading to agammaglobulinemia. Similar to Btk, BLNK is a protein involved in pre-B cell signal transduction, and defects in BLNK lead to agammaglobulinemia by this mechanism (Minegishi et al. 1999). Leucine-rich repeat-containing eight gene codes for a protein of unknown function. However, a truncated version of the protein results in arrest at the pre-B cell stage and agammaglobulinemia by mechanisms yet to be elucidated (Sawada et al. 2003; Table I).

*Hyper IgM syndrome*

Both activation-induced cytidine deaminase (AICD) defect and uracil nucleoside glycosylase (UNG) defect are part of the so-called hyper IgM syndrome. Although we will focus primarily on the autosomal group in this section, some of the immunology described applies to all types. In addition, a third autosomal recessive form of hyper IgM syndrome will be discussed in the combined immunodeficiency section along with the X-linked form given their closely related molecular defects. As the name suggests, this syndrome consists of very low levels of IgG, IgA, and IgE but normal or elevated levels of polyclonal IgM (Levitt et al. 1983). All types of hyper IgM syndrome are due to problems with Ig gene class-switching and somatic hypermutation (Levitt et al. 1983). B cells first produce IgM and IgD during a primary antibody response (Bonilla and Geha 2003). Class switching refers to the process whereby the B cell Ig genes are rearranged as the immune response progresses. This gene rearrangement and the resultant "class switch" to production of IgG, IgA, and IgE is vital for resistance to bacterial infections and requires interaction between T and B cells and enzyme-driven modifications of B cell genetic material. When the T–B cell interaction goes awry (due to defects in CD40 Ligand or CD40 that are discussed further in the X-linked hyper IgM syndrome section), class switching and somatic hypermutation do not occur and the hyper IgM phenotype is seen. Somatic hypermutation refers to the accumulation of point mutations in the Ig-gene variable regions such that the accumulated mutations increase the antibody's affinity for the antigen (Bonilla and Geha 2003). In the autosomal recessive hyper IgM syndromes, the problem lies in the nucleotide-editing enzymes AICD or UNG (Levitt et al. 1983, Revy et al. 2000, Durandy et al. 2003). These enzymes are only present in the germinal center B cells, and defects in either disrupt B-cell development and antibody production. Unlike the hyper IgM syndromes due to defects in CD40–CD40L interactions, the hyper IgM syndromes due to these enzyme defects are associated with defective formation of germinal centers (Durandy et al. 2003). This disordered B cell development leads to lymphoid hyperplasia, which is not seen with the types discussed later. Patients with these enzyme defects have severe hypogammaglobulinemia and have infections similar to those of patients with XLA. T cell numbers and function are normal in these two diseases. The treatment of choice is IVIG (Revy et al. 2000; Table I).

*Common variable immunodeficiency (CVID) associated with inducible T cell costimulator (ICOS) deficiency or transmembrane activator and calcium modulator and cyclophilin ligand interactor (TACI) deficiency*

CVID is a clinically defined disease characterized by low IgG, possibly low IgA, and a significant defect in specific antibody formation when challenged with vaccines or natural pathogens (Conley et al. 1999, Bonilla and Geha 2003, Goldacker and Warnatz 2005). Although the vast majority of cases of CVID are of unknown genetic and molecular basis, a minority of patients with CVID have been identified that have genetic mutations.

*ICOS* is a gene that codes for ICOS, a T cell surface protein that interacts with ICOS ligand found on B cells (Grimbacher et al. 2003, Salzer et al. 2004). Without this interaction, patients display panhypogammaglobulinemia, poor specific antibody production, and a clinical phenotype meeting criteria for CVID (Bonilla and Geha 2006). Features of CVID such as splenomegaly, sarcoid-like granulomatous disease, and autoimmune disease are also seen with ICOS defects (Vihinen 2004). ICOS seems to be necessary for T cell-dependent late B cell differentiation, class-switching and formation of memory B cells (Vihinen 2004). This disorder has an onset delayed until late childhood or adulthood. Only 9 of 226 patients with CVID screened for ICOS defects have been found to have the ICOS mutations, each of them is from the Black Forest region of Germany, and each carries the same deletion (Salzer et al. 2004, Bonilla and Geha 2006).

Seventeen of 181 patients with CVID and one of 16 with selective IgA deficiency have had mutations in the gene encoding the B cell surface protein called transmembrane activator and calcium modulator and cyclophilin ligand interactor (TACI) (Salzer and Grimbacher 2005, Salzer et al. 2005). TACI interaction with B-cell activating factor (BAFF) and a proliferation-inducing ligand (APRIL) on macrophages and dendritic cells is important for activation of B cells and class switching (Salzer and Grimbacher 2005, Salzer et al. 2005). Except for the single patient with selective IgA deficiency, these patients display panhypogammaglobulinemia, autoimmunity, lymphoproliferation with hepatosplenomegaly and cancer, and inadequate antibody response to infectious or vaccine challenge (Bonilla and Geha 2006; Table I).

*Common variable immunodeficiency (CVID) (due to unknown defect)*

Specific molecular defects have not been identified in the vast majority of patients with CVID. CVID is a syndrome with highly variable presentation and includes a heterogeneous group of disorders. It is typically defined by poor antibody responses to infection/vaccines with low IgG, usually low IgA, and sometimes low IgM serum levels. Onset can be at any age but peaks in early childhood and early adulthood (Vihinen 2004). Onset is usually later than that of XLA, and both sexes are equally affected (Buckley 2003a). The types of pathogens these patients are infected with tend to be the same as those with XLA. Infections of the gastrointestinal and respiratory tracts are common, sometimes leading to chronic sinusitis or bronchiectasis. Giardiasis is also common (Buckley 2003a). Associated problems may variably include autoimmune disorders such as hemolytic anemia, thrombocytopenia, seronegative arthritis, sicca, vitiligo, thymoma, alopecia areata, pernicious anemia, and vasculitis (Buckley 2003a). Thyroid disease is a frequent finding (Buckley 2003a). Benign or malignant thymoma in CVID patients may lead to myasthenia gravis or hematologic disease (Vihinen 2004). A sprue-like syndrome is also found (Goldacker and Warnatz 2005). Lymphoid proliferation is seen in about a third of patients, while the chance of lymphoma is increased by more than 300-fold (Bonilla and Geha 2003, Buckley 2003a). Tonsils and peripheral lymph nodes may be normal or enlarged, with splenomegaly occurring in a quarter of cases (Buckley 2003a). Other malignant complications include gastric carcinoma. A unique manifestation is nodular hyperplasia in the bowel (Vihinen 2004). About 10% of patients have asthma and allergic rhinitis without antigen-specific IgE (Buckley 2003a). Non-caseating granulomatous disease and amyloidosis are also seen (Buckley 2003a, Morimoto and Routes 2005).

Despite the hypogammaglobulinemia, CVID patients typically have normal numbers of blood T and surface Ig-bearing B cells (Buckley 2003a). As with most predominantly antibody-deficient PID patients, CVID patients normally handle viral and fungal infections. CVID has usually been thought to be due to B cell defects, and inability of CVID B-cells to differentiate into plasma cells despite stimulation and the presence of normal T cells *in vitro* support this belief (Cunningham-Rundles 1989). Additional support of intrinsic B cell etiologies include lack of L-selectin on B cells and lack of proper protein kinase C activation and translocation in stimulated CVID B cells *in vitro* (Kaneko et al. 1996, Zhang et al. 1996). However, recent data suggests that inadequate signaling from T cells (cellular defects) could be contributing to the B cell differentiation problems in CVID (Buckley 2003a). Specifically, some patients have abnormal CD4 T cell differentiation or abnormal T cell function, and CVID B cells can be stimulated to isotype switch and produce Ig by providing artificial T cell help (Spickett et al. 1990, Nonoyama et al. 1993, Farrant et al. 1994, Farrington et al. 1994).

The pathogenesis of CVID is unknown. It has been speculated that a common genetic problem may result in IgA deficiency (IGAD) and CVID based on the facts that first-degree relatives of CVID patients often have selective IGAD and that some IGAD patients become panhypogammaglobulinemic (Hammarstrom et al. 2000). Additional support for this includes the high prevalence of autoimmune and malignant disease in both disease groups (Hammarstrom et al. 2000). Although particular MHC haplotypes have been found to be abnormally frequent in patients with CVID and IGAD, environmental factors such as drugs like phenytoin may play a triggering role in patients with appropriate genetic susceptibility (Ashman et al. 1992, Buckley 2003a). As other PIDs may initially be diagnosed as CVID, it is important to consider performing genetic screens in male CVID patients for X-linked lymphoproliferative disorder, XLA, X-linked hyper IgM, AICD defects, and CD40 defect-related autosomal recessive hyper IgM syndrome (Buckley 2003a). Females with CVID should be screened for only the last two defects.

IVIG and aggressive treatment of infections are the main treatments for CVID (Eisenstein and Sneller 1994). Early diagnosis and treatment may prevent complications such as bronchiectasis. As CVID patients with low IgA levels may have anti-IgA antibodies that can cause anaphylaxis when given IgA-containing IVIG, caution and screening for anti-IgA antibodies are warranted before starting such IVIG (Burks et al. 1986). If these antibodies are detected, IVIG containing low levels of IgA may be used cautiously. CVID patients have a reasonably good prognosis if severe autoimmune or malignant disease does not develop (Buckley 2003a; Table I).

*Selective IgA deficiency (IGAD)*

Selective IGAD is the most common PID, with an incidence from 1 in 333 to 1 in 700 (Cunningham-Rundles 2001). It is defined by serum IgA levels of 10 mg/dl or less with normal concentrations of other Ig isotypes (Buckley 2003a). Many patients with IGAD are asymptomatic, but those with symptoms are prone to infectious complications involving mucosa (gastrointestinal, respiratory, urogenital) and pathogens common to other humoral PIDs. Viral infection is not common. Like CVID, IGAD may have associated autoimmune, autoantibody, collagen vascular, and malignant disease (Vihinen 2004). Atopic symptoms with specific IgE are common (Bonilla and Geha 2003).

Immunologically, in addition to the IgA deficiency, some may have IgG2 subclass deficiency with elevated IgM (Sandler et al. 1996). IgE and other antibodies against IgA may be present in nearly half of IGAD patients (Clark et al. 1983, Sandler et al. 1995, Sandler and Zantek 2004, Sandler 2006). These anti-IgA antibodies can cause severe or fatal anaphylaxis if IgA-containing blood products (such as IVIG) are infused into IGAD patients. Therefore, IGAD patients should receive blood products from other IGAD patients or normal donor red blood cells after five washes (Buckley 2003a).

The failure of terminal differentiation in IgA-positive B cells in IGAD is of unknown etiology, but genetic studies have suggested that HLA-DQ/DR is the major IGAD1 locus (Vihinen 2004). The pathophysiologic mechanisms causing disease remain unclear. Inheritance patterns are variable, both sexes are equally affected, and drug triggers are suspected to facilitate expression (Buckley 2003a). As mentioned above, a common genetic relationship with CVID is also postulated (Hammarstrom et al. 2000).

Treatment of IGAD consists of antibiotics for infections that develop. IVIG is not appropriate given the risk of anaphylaxis and since most IGAD patients do not lack IgG, which is what IVIG provides (Cunningham-Rundles 2001). Prognosis of asymptomatic patients is excellent. Symptomatic children may display resolution of the disease, while adults tend to have persistent disease that may develop into CVID in some (Hammarstrom et al. 2000, Buckley 2003a; Table I).

*Specific antibody deficiency with normal immunoglobulins (SADNI)*

Specific antibody deficiency with normal Igs (SADNI) is a relatively common PID, representing 23% of PID cases in one tertiary center (Javier et al. 2000). It is of unknown etiology and characterized by normal amounts of Ig isotypes and subtypes but an impaired ability to make specific antibody, especially against polysaccharides (Antall et al. 1999; Table I).

*Immunoglobulin G subclass deficiency (IGGSD)*

IGGSD is another PID with unknown etiology and characterized by normal total IgG with low or nonexistent levels of one or more of the IgG subclasses (IgG1, IgG2, IgG3, or IgG4). Most patients with IGGSD are asymptomatic, but some do get recurrent sinopulmonary infections from encapsulated bacteria (Morell 1994). Although the IgG2 subclass makes up most of the antibodies against polysaccharides, the clinical importance of IgG2 in preventing disease is not clear, as there are patients with normal IgG2 levels who cannot form antibodies against polysaccharides and those with low IgG2 levels who can (Shackelford et al. 1990a,b, Shackelford 1993, Alyanakian et al. 2003). In some children with infections with low IgG2 levels, a more thorough immunologic workup up reveals a broader pattern of immune dysfunction than that of children with asymptomatic IgG2 deficiency (Shackelford et al. 1990b). Experts suggest that IgG subclass measurement and deficiency is not of clinical utility unless there is a corresponding deficiency in production of specific antibodies to a broad array of protein and polysaccharide antigens (Buckley 2003a). IVIG use in IGGSD patients not meeting the latter criterion is not appropriate. Specific infections may be treated with appropriate antibiotics, and evidence of specific antibody production defects should be sought (Table I).

*Transient hypogammaglobulinemia of infancy (THI)*

THI is defined as a low level of IgG associated with recurrent bacterial and viral infections which resolves by age four (Rosefsky 1990, Kilic et al. 2000, Dogu et al. 2004). Most patients make normal specific antibodies, and serious infections are uncommon. THI is not an indication for IVIG (Table I).

*Immunodeficiency, centromeric instability, facial anomaly syndrome (ICF syndrome)*

ICF syndrome is a rare autosomal recessive syndrome associated with mutations in the DNA methyltransferase 3B gene in 75% of cases (Blanco-Betancourt et al. 2004). Patients have variable hypogammaglobulinemia but typically have profound reduction or absence of two or more Ig isotypes (Vihinen 2004). This leads to severe immunodeficiency and death due to infection often before adulthood (Vihinen 2004). Peripheral blood B cells are limited to naïve B cells, which also often express autoreactive heavy chain variable regions (Blanco-Betancourt et al. 2004). This is thought to suggest abnormal B cell negative selection (Blanco-Betancourt et al. 2004). *In vitro* studies show increased apoptosis of these B cells. Some cases also display impaired cellular immune function, neurologic, and intestinal dysfunction (Vihinen 2004). The facial anomalies include low-set ears, epicanthal folds, flat nasal bridge, hypertelorism, and macroglossia (Bonilla and Geha 2006; Table I).

### Cellular PIDs

Cellular PID is defined as defective T cell or NK cell function with normal or largely normal humoral immunity. Infections in patients with cellular PIDs tend to be from viral, fungal, or opportunistic organisms such as mycobacteria (Figure 1). PIDS with primarily phagocyte defects, which one might also consider "cellular", are often grouped separately. This convention will be followed herein. However, since defects of the interferon-γ/IL-12 axis may affect T cells, NK cells, and traditional phagocytes such as monocytes and macrophages, these disorders and their role in T cell and NK predominant disease will be discussed. Table II lists these various disorders, and additional description of significant disorders is noted in sections below. Management of cellular PIDs of significant severity, as well as cellular deficiency that is part of combined immunodeficiency, is limited in terms of effective therapeutic options. The treatment of choice to correct the cellular deficiency is usually a BMT.

*Defects in the interferon-**γ**/IL-12 axis: IL-12 p40 subunit deficiency; IL-12 receptor **α**1 chain deficiency; IFN-**γ** receptor **α** chain deficiency; IFN-**γ** receptor **α** chain deficiency; and signal transducer and activator or transcription 1 (STAT-1) deficiency*

Interferon-γ (IFN-γ) is vital in activating mononuclear cell cytotoxic pathways needed to control intracellular pathogens such as *Salmonella* and mycobacteria (Bonilla and Geha 2003). IL-12 is the main stimulus for IFNγ production by $T_H1-T$ cells and NK cells (Doffinger et al. 1999). Cellular PID due to mutations in components of IL-12, the IL-12 receptor, and the IFN-γ receptor has been reported. The same is true for defects in signal transducer and activator of transcription (STAT) 1, as this molecule allows signaling via the IFN-γ receptor. Partial IFN-γ receptor, IL-12, and IL-12 receptor deficiency may respond to subcutaneous injections of IFN-γ (Bonilla and Geha 2003; Table II).

Deficiency of the p40 subunit of IL-12 is an autosomal recessive defect that usually results in mild infections due to intracellular organisms (Vihinen et al. 2001, Notarangelo et al. 2004). The abnormal IL-12 production prevents normal IFN-γ secretion. The macrophage is the main cell affected. A similar clinical phenotype results from defective IL-12R β1 chain, however this disorder primarily affects lymphocytes and NK cells (Vihinen et al. 2001, Notarangelo et al. 2004).

Defects in the α or β chain of the IFN-γ receptor similarly lead to *Salmonella* and mycobacterial infections. These disorders affect both macrophages and lymphocytes, since both are dependent on the Il-12/IFN-γ pathway to fight intracellular infection. With the IFN-γ receptor defects, partial defects cause mild disease, while defects resulting in complete absence of either the α or β chain of the IFN-γ receptor lead to severe infections (Vihinen et al. 2001, Notarangelo et al. 2004). The α chain defect is also characterized by atopy, glomerulonephritis, vasculitis, and a positive rheumatoid factor (Vihinen et al. 2001, Notarangelo et al. 2004).

After IFN-γ attaches to the IFN-γ receptor on macrophages and lymphocytes, the signal is transduced by a transcription factor called STAT1. STAT1 binds response elements within the nucleus to trigger production of inflammatory mediators of cellular cytotoxicity (Vihinen et al. 2001). Defects in STAT1 cause increased propensity for infection due to mycobacteria and Salmonella, but not viruses (Vihinen et al. 2001, Notarangelo et al. 2004). There are both autosomal dominant and recessive forms of this disorder. As in IFN-γR deficiency, atopy, glomerulonephritis, vasculitis, and a positive rheumatoid factor are also found in this disorder and are a marker of immune dysregulation (Vihinen et al. 2001).

Table II. Cellular PIDs.

| Disorder | Presumed pathogenetic mechanism | | | Classic/associated features | Affected cell (s) | B cells # (blood) | Serum Ig | Inheritance |
|---|---|---|---|---|---|---|---|---|
| | Abnormal gene | Abnormal genetic locus | Abnormal gene product | | | | | |
| IFN-γ/IL-12 axis | | | | | | | | |
| IL-12 p40 subunit deficiency | *IL12B* | 5q31.1–q33.1 | IL-12 p40 | Mycobacteria and Salmonella susceptibility, mild symptoms | M | N | N | AR |
| IL-12 receptor (IL-12R) β1 chain deficiency | *IL12RB1* | 19p13.1 | IL-12R β1 | Mycobacteria and Salmonella susceptibility, mild symptoms | L + NK | N | N | AR |
| IFN-γ receptor (IFNγR) α chain deficiency (IFNγR1 deficiency) | *IFNGR1* | 6q23–24 | IFN-γR α | Mycobacteria and Salmonella susceptibility, mild if partial defect, severe if full defect; atopy, glomerulonephritis, vasculitis, rheumatoid factor | M + L | N | N | AR, AD |
| IFN-γ receptor (IFNγR) β chain deficiency (IFNγR2 deficiency) | *IFNGR2* | 21q22.1–q22.2 | IFN-γR β | Mycobacteria and Salmonella susceptibility, mild if partial defect, severe if full defect | M + L | N | N | AR |
| Signal transducer and activator of transcription 1 (STAT-1) deficiency | *STAT1* | 2q32.2–q32.3 | STAT-1 | Mycobacteria and Salmonella susceptibility | M + L | N | N | AR, AD |
| NK cell defects | | | | | | | | |
| CD16 (FcγRIIIa) deficiency (NK deficiency) | *FCGR3A* | 1q23 | FcγRIIIa | Viral infections, abnormal response to BCG vaccine | NK mainly | N | N | ? |
| Cellular PIDs with unknown molecular basis | | | | | | | | |
| Chronic mucocutaneous candidiasis (CMCC) | ? | ? | ? | Fungal (especially *Candida albicans*) infection, possible endocrinopathy or thymoma, possible bacterial and viral infection | ? | N | N | Varies |
| Idiopathic $CD4^+$ T lymphocytopenia | ? | ? | ? | Opportunistic infections, autoimmune disease, hematologic malignancy; HIV and viral studies negative | ↓ $CD4^+$ T cell | N | N | ? |
| Isolated NK cell defects | ? | ? | ? | NK cell number or function deficit; B and T cells normal; predisposed to herpesvirus or papillomavirus infection; important to rule out other PIDs associated with NK cell defects | NK | N | N | ? |

Data abstracted from de Vries et al. 1996, Anonymous 2000, Lilic 2002, Orange 2002, Buckley 2003a, Chapel et al. 2003, Notarangelo et al. 2004, Vihinen 2004, Bonilla and Geha 2006. Abbreviations: AD, Autosomal dominant; AR, Autosomal recessive; XL, X-linked; M, monocyte/macrophage; L, lymphocyte; NK, Natural killer; ↓, decreased; ↓↓, profoundly decreased; ↑, increased; N, normal; for Serum Ig Column, “All” refers to all isotypes.

NK cell defects

*CD 16 deficiency (FcγRIIIa deficiency, NK deficiency)*

Recurrent viral infection with a cellular PID phenotype has been reported in a single boy who had a mutation in CD16, also known as FcγRIIIa (de Vries et al. 1996). CD16 is part of the FcγRIII found on NK cells as well as macrophages and some T cells. The receptor allows NK cells to phagocytose organisms or cells coated with IgG in the absence of MHC (antibody-dependent cellular cytotoxicity). The mutation disrupts NK cell function and is associated with NK cytopenia. The patient also had problems after BCG vaccination (Table II).

*Natural killer cell deficiency (due to unknown defect)*

Isolated defects in NK cell numbers and/or function due to poorly characterized abnormalities have been reported and often include severe herpesvirus infection (Orange 2002). If such disorders are suspected, it is important to rule out other PIDs associated with NK defects, including XLA, Chediak-Higashi syndrome, severe combined immunodeficiency disorder (SCID), Wiskott–Aldrich syndrome, and nuclear factor κB essential modulator deficiency, and of course CD16 deficiency (Bonilla and Geha 2006; Table II).

Other cellular PIDs

*Chronic mucocutaneous candidiasis (due to unknown defect)*

CMCC refers to a heterogeneous group of diseases rather than a single PID. CMCC is considered secondary to abnormal cellular immunity, though the cause is not known in most cases apart from the Autoimmune polyendocrinopathy–candidiasis–ectodermal dystrophy (APECED) subset. Patients have recurrent and difficult to treat infections of the skin, mucous membranes, or nails with fungal organisms, particularly *Candida albicans* (Lilic 2002). There may be associated endocrinopathy (see APECED above) or thymoma (Vihinen 2004). Although it is felt to be a cellular defect, some patients have bacterial infections in addition to fungal, viral, *Toxoplasma*, and mycobacterial disease. Animal and human studies suggest decreased amounts of Th1 cytokines in these patients (Lilic 2002). Treatment includes topical and oral antifungal agents. Management also requires regular screening for endocrinopathy such as hypothyroidism, adrenal insufficiency, and hypoparathyroidism (Vihinen 2004; Table II).

*Idiopathic $CD4^+$ T lymphocytopenia*

Idiopathic $CD4^+$ T lymphocytopenia is a rare PID resembling HIV infection. All known tests for this and other viruses are negative, however. CD4 counts are less than 300 cells/$mm^3$, and patients develop opportunistic infection, autoimmunity, as well as hematological malignancies (Bonilla and Geha 2006; Table II).

### Combined PIDs

Combined PIDs are defined by abnormal cellular immunity combined with abnormal humoral immunity. The latter may occur in the form of normal or elevated numbers of B cells that do not function well (as in $T^-B^+$ SCID) or in the form of significantly reduced or absent B cells (as in $T^-B^-$ SCID). As expected, combined PIDs complications include bacterial, viral, fungal, mycobacterial, and opportunistic infections. Chronic diarrhea with failure to thrive is commonly seen, as are recurrent sinopulmonary infections and systemic infections (Bonilla and Geha 2003). Severe combined immunodeficiency (SCID) is the term used by most to refer to combined PIDs with severe or absent T cell function associated with humoral immunodeficiency. When T cell function is low but not absent, some experts refer to this as "combined immunodeficiency"(CID). Examples of CID include purine nucleoside phosphorylase deficiency, ataxia–telangiectasia, and cartilage–hair hypoplasia. The classic example of SCID is X-linked SCID. Given the variability of presentations of a particular PID with humoral and cellular immunodeficiency, some authors do not always make a distinction between CID and SCID. In addition to the combined PIDs with known molecular defects, there are SCIDs and CIDs with unknown molecular defects.

Management of combined PIDs requires specific and sometimes prophylactic antibiotic use and vaccination with appropriate non-live vaccines. IVIG is indicated to treat the humoral defect, but is not sufficient to control combined disease. SCID patients have been treated with BMTs for many years. Success rates vary from 50 to 100% based on the age at BMT, donor marrow type, and the particular type of SCID (Bonilla and Geha 2003). When BMT is needed, the treatments of choice are an HLA-identical related donor or HLA-haploidentical related donor (Buckley 2003a). Graft vs. host disease (GVHD) is prevented while using haploidentical donor marrow by depleting mature T cells from the donor marrow before transplant. This has allowed successful BMT in hundreds of infants with SCID who did not have an HLA-identical marrow donor (1993, Buckley et al. 1999, Buckley 2003b). HLA-identical BMT is an option for patients with partial DiGeorge syndrome, while complete DiGeorge syndrome requires transplant of HLA-matched fetal thymic epithelial cells for cure (Buckley 2003a, Cleveland 1975, Thong et al. 1978). Although two forms of SCID, X-linked SCID and adenosine deaminase (ADA) deficiency have been successfully treated with gene therapy, leukemia in several patients has brought this form of treatment under closer scrutiny (Aiuti et al. 2002, Aiuti 2004, Gaspar et al. 2004). Table III as well as the sections below describe combined PIDs.

### Severe combined immunodeficiency diseases (SCIDs)

### *Common γ chain deficiency (X-linked SCID, SCID-X1, γc SCID)*

SCIDs represent a large and ever-expanding group of PIDs, many with known molecular defects. As the name suggests, SCIDs display the most severe cellular immune dysfunction, sometimes with complete absence of functional lymphocytes. The prototypical SCID is common γ chain deficiency (γc SCID). As such, much of what is described about it applies to the other SCIDs as well. Like the other members of this subgroup, γc SCID is a T−B+ SCID, suggesting that B cells are usually present, but are not normal functionally. γc SCID is the most common SCID and X-linked (Bonilla and Geha 2003). γc SCID should be considered a pediatric emergency that is fatal if untreated. Patients present within the first few months of life with recurrent sinopulmonary, skin infections, and diarrhea. As with most SCIDs, the pathogens include bacteria, viruses, mycobacteria, and opportunistic organisms. Infections can be fatal. GVHD may also occur due to maternal T cells that entered the patient during gestation or from immunocompetent T cells present in donated bone marrow or non-irradiated blood products (Buckley 2003a). Affected infants have low lymphocyte counts and abnormal lymphocyte proliferation to stimuli (Uribe and Weinberg 1998). Absolute lymphopenia from cord blood of newborns is defined as 2000–11,000/mm$^3$, while that of six month old infants is below 4000 (Buckley 2003a). There is a profound decrease or total absence of T cells. B cell numbers are normal or increased but specific antibody responses are absent. Serum Ig levels are low. NK cell number and function are also low. As with most SCID patients, γc SCID infants have small, histologically abnormal thymuses that are, however, able to educate T cells. Tonsil, adenoid, and peripheral lymphoid tissues are small or absent (Buckley 2003a).

The defective gene in γc SCID encodes for an abnormal or absent cytokine receptor γ chain, a protein that is a part of the receptor complex for multiple cytokines including IL-2, IL-4, IL-7, IL-9, IL-15, and IL-21 (Asao et al. 2001). The defect leads to widespread problems in cytokine signaling and the immunologic defects discussed.

Treatment for γc SCID and most SCIDs is bone marrow transplantation. IVIG does not prevent progression of the disease, which is fatal by the first or second year of life if not treated (Buckley 2003a). Although gene therapy using a retrovirus to insert a normal gene into the host has been successful, occurrences of leukemia with such treatment have been reported (Gaspar et al. 2004). Successful treatment relies heavily on early diagnosis, which can be facilitated by white blood cell counts with manual differential on cord blood (Buckley 2003a, Gaspar et al. 2004). This test is not routine (Table III).

### *Janus kinase 3 deficiency (Jak3 deficiency)*

Janus Kinase 3 (Jak3) is a signaling molecule associated with the common γ chain (Rane and Reddy 2000). Deficiency of Jak 3 produces a clinical and immunologic phenotype similar to γc SCID (Table III).

### *CD3δ deficiency*

The T cell receptor complex consists of two groups of proteins. The first, called $T_i$ is a heterodimer (αβ or γδ) that has the variable, antigen-binding site. The second is the invariant protein complex called CD3, which is comprised of one γ, one δ, two ϵ, and two ζ subunits. CD3 transduces the signal generated by antigen binding to the antigen-binding site of the $T_i$. Interestingly, CD3δ deficiency results in T cell numbers in the blood to be very low or absent, while deficiencies in CD3ϵ or CD3γ result in normal numbers of circulating T cells that are dysfunctional (Buckley 2003a). CD3δ deficiency thus produces a SCID, while the latter defects produce a CID that usually is mild (Notarangelo et al. 2004) (Table III).

### *IL-7 receptor α deficiency*

The IL-7 receptor (along with IL-7) is important in T cell function. However, the fact that IL-7Rα deficiency is associated with normal numbers of NK cells suggests that this cytokine pathway is not essential for NK development (Buckley 2003a). Another feature distinguishing this disorder from γc SCID is that BMT corrects the B cell function in IL7Rα deficiency (Table III).

### *Il-2 receptor α deficiency*

The IL-2 cytokine pathway is needed for T cell development and function. Mutation in the IL-2 receptor's α chain (CD25) results in a clinical phenotype very similar to the prototypical $T^-B^+$ SCID, X-linked SCID (Bonilla and Geha 2003). The single patient described with this defect had lymphocytic infiltration of her organs. The immunophenotype is characterized by normal B cell numbers in the blood (Vihinen 2001, 2004). There are low T cell numbers due to abnormal thymocyte

Table III. Combined PIDs.

| Disorder | Abnormal gene | Abnormal genetic locus | Abnormal gene product | Classic/associated features | T Cell # (blood) | B Cells # (blood) | Serum Ig | NK Cell # (blood) | Inheritance |
|---|---|---|---|---|---|---|---|---|---|
| $T^-B^+$ SCIDs | | | | | | | | | |
| X-linked SCID (γc SCID) | *IL2RG* | Xq13.1 | Common γ chain | Bacterial, viral, fungal, mycobacterial, opportunistic infections, diarrhea, FTT, possible cure with BMT but B cell dysfunction persists, gene therapy exists but leukemia in some, small/absent lymphoid tissue, GVHD risk | ↓↓ | N or ↑ | ↓ | ↓↓ | XL |
| Janus kinase-3 (Jak3) deficiency | *JAK3* | 19p13.1 | Jak3 | Same as γc SCID, (atypical cases may have T cells) | ↓↓ | N or ↑ | ↓ | ↓↓ | AR |
| IL-7 receptor α (IL7Rα) deficiency | *IL7R* | 5p13 | IL7R α | Same as γc SCID except BMT restores B and T cell immunity, $T^-B^+NK^+$ SCID | ↓↓ | N or ↑ | ↓ | N | AR |
| Il-2 receptor α (IL2Rα) deficiency (CD25 deficiency) | *IL2RA* | 10p15–p14 | IL2Rα | Same as γc SCID, extensive lymphocytic infiltration of organs, absence of IL2Rα on thymic epithelial cells impairs T cell differentiation, 1 case | ↓ | N | IgA ↓, others N or ↑ | – | AR |
| CD45 deficiency | *PTPRC* | 1q31–q32 | CD45 | Typical combined PID infections, normal γδ T cells | ↓↓ | N | ↓ | – | AR |
| CD3δ deficiency | *CD3D* | 11q23 | CD3δ | Typical combined PID infections; may still have thymic shadow but thymocytes fail to mature | ↓↓ | N | ↓ | N | AR |
| Winged Helix Nude (WHN) deficiency | *FOXN1* | 17q11–q12 | WHN gene product | Alopecia, nails pitted and ridged, typical combined PID infections, thymic epithelium abnormal, CD8 numbers normal | ↓↓ | N | ↓ | – | AR |
| Immunodeficiency with thymoma | ? | ? | ? | Thymomas usually benign, may have eosinophilia/eosinopenia, anemia, agranulocytosis, thrombocytopenia, pancytopenia | ↓ | N | ↓ | – | ? |
| $T^-B^-$ SCIDs | | | | | | | | | |
| Adenosine deaminase (ADA) deficiency | *ADA* | 20q13.2–q13.11 | ADA | Typical combined PID infections, profound lymphopenia, NK function normal, BMT restores B and T cell immunity | Progressive ↓ | Progressive ↓ | ↓ | ↓ (but functional) | AR |
| Recombinase activating gene (RAG) deficiencies: RAG1 deficiency, RAG2 deficiency | *RAG1/2* | 11p13 | RAG1/2 | $T^-B^-NK^+$ SCID, NK cell is main cell in circulation, deficiency due to defective VDJ recombination; usual defect causing Omenn syndrome (see below) | ↓↓, but may have oligoclonal T cells | ↓↓ | ↓ but ↑ IgE | N | AR |
| Artemis deficiency | *DCCRE1C* | 10p13 | Artemis | $T^-B^-NK^+$ SCID, radiation sensitivity, deficiency due to defective VDJ recombination; sometimes associated with Omenn's syndrome | ↓ | ↓ | ↓ | N | AR |
| Reticular dysgenesis | ? | ? | ? | Absent T and B cells and granulocytes, deafness, thrombocytopenia | ↓↓ | ↓↓ | ↓ | – | Likely AR |

Table III – *continued*

| Disorder | Abnormal gene | Abnormal genetic locus | Abnormal gene product | Classic/associated features | T Cell # (blood) | B Cells # (blood) | Serum Ig | NK Cell # (blood) | Inheritance |
|---|---|---|---|---|---|---|---|---|---|
| Other SCIDs | | | | | | | | | |
| Selective IL-2 production defect | ? | ? | ? | Il-2 gene present but Il-2 selectively not produced, only 2 cases | N | – | – | – | ? |
| Multiple lymphokine production defects | ? | ? | ? | IL-2, IL-4, IL-5, IFN-γ, and TNF-α may be lacking, only 4 cases, may be due to abnormal NFAT binding to lymphokine promoters on gene | N | – | – | – | ? |
| CIDs | | | | | | | | | |
| X-linked hyper IgM syndrome | *TNFSF5* | Xq26.3–q27.1 | CD40L | Neutropenia, autoimmune cytopenias, thrombocytopenia, hemolytic anemia, hepatoma and other cancers, typical combined PID infections, deficiency due to abnormal CD40L/CD40 interaction | N | Only IgM or IgD-bearing cells | IgM ↑ or N, rest ↓ | – | XL |
| CD40 deficiency hyper IgM syndrome | *TNFRSF5* | 20q12–q13.2 | CD40 | Neutropenia, hemolytic anemia, hepatic and gastrointestinal involvement, opportunistic infections, deficiency due to abnormal CD40L/CD40 interaction | N | Only IgM or IgD-bearing cells | IgM ↑ or N, rest ↓ | – | AR |
| Purine nucleoside phosphorylase (PNP) deficiency | *NP* | 14q13.1 | PNP | AIHA, neurologic symptoms, T cell deficit due to toxic metabolites from enzyme deficiency | ↓ progressive | N | N or ↓ | ↑ | AR |
| Omenn syndrome | *RAG1* or *2* | 11p13 | RAG1 or 2 | hepatosplenomegaly, hypereosinophilia, erythroderma, desquamation, and diarrhea, deficiency due to defective VDJ recombination | N #, oligoclonal | Usually ↓ | Most ↓ but ↓ IgE | NK | AR |
| MHC I deficiencies: | | | | | | | | | |
| Transporter associated protein (TAP) 1 deficiency | *TAP-1* | 6p21.3 | TAP-1 | Vasculitis, relatively mild PID; if defect due to tapasin, low level of MHC I may be expressed | CD8 ↓, N CD4 | N | N | – | AR |
| TAP-2 deficiency | *TAP-2* | 6p21.3 | TAP-2 | | | | | | |
| TAP-binding protein (Tapasin) deficiency | *TAPBP* | 6p21.3 | tapasin | | | | | | |
| MHC II deficiencies: | | | | | | | | | |
| RFX5 deficiency | *RFX5* | 1q21 | RFX5 | More severe than MHC I deficiency but less than SCID | CD4 ↓, N CD8 | N | N or ↓ | – | AR |
| RFXAP deficiency | *RFXAP* | 13q14 | RFXAP | | | | | | |
| RFXANK deficiency | *RFXANK* | 19p12 | RFXANK | | | | | | |
| CIITA deficiency | *MHC2TA* | 16p13 | MHCIITA | | | | | | |
| Zeta-associated protein 70 (ZAP-70) deficiency | *ZAP70* | 2q12 | ZAP-70 kinase | Majority in Mennonites, may be fatal but less severe and later presentation than SCID, abnormal thymic selection | CD8 ↓, N CD4 | N | N or ↓ or ↑ | N | AR |
| p56 Lck deficiency | *LCK* | 1p35–p34.3 | p56 Lck | T cell activation defect | CD4 ↓ | N | ↓ | N | ? |

Table III – *continued*

| Disorder | Abnormal gene | Abnormal genetic locus | Abnormal gene product | Classic/associated features | T Cell # (blood) | B Cells # (blood) | Serum Ig | NK Cell # (blood) | Inheritance |
|---|---|---|---|---|---|---|---|---|---|
| CD3$\epsilon$ deficiency, CD3$\gamma$ deficiency | *CD3E*, *CD3G* | 11q23, 11q23 | CD3$\epsilon$, CD3$\gamma$ | T cell activation defect | N but dys-functional | N | N | – | AR |
| CD8 deficiency due to CD8a gene mutation | *CD8A* | 2p12 | CD8 | 1 case | CD8 ↓, N CD4 | N | N | N | AR |
| DNA repair defects | | | | | | | | | |
| Ataxia-telangiectasia (AT) | *ATM* | 11q22–q23 | ATM protein | Cerebellar ataxia, oculocutaneous telangiectasia, Sensitivity to ionizing radiation, 30% develop cancer, growth retardation, sexual immaturity, | ↓ | N | Often ↓ IgA, IgE, IgGs; ↑ IgM; varies | – | AR |
| Ataxia–telangiectasia-like syndrome | *MRE11A* | 11q21 | MRE11a protein | Moderate ataxia, severely increased radiosensitivity, otherwise similar to but milder than AT | ↓ | N | See AT | – | AR |
| Nijmegan breakage syndrome (NBS) | *NBS1* | 8q21 | NBS1 protein | Short stature, progressive microcephaly/ cognitive decline, lymphoma, radiosensitivity, chromosomal instability, abnormal facies with age, infections (pulmonary), ovarian failure, irregular skin pigmentation | ↓ | N | See AT | – | AR |
| DNA ligase I deficiency | *LIG1* | 19q13.2–q13.3 | DNA ligase 1 | 1 case, sun sensitivity, short stature, lymphoma, sinopulmonary infections, developmental delay, sexual immaturity, similar to Bloom syndrome | – | – | – | – | AR |
| DNA ligase IV deficiency | *LIG4* | 13q22–q34 | DNA ligase 4 | Microcephaly, facial dystrophy, radiosensitivity, clinically similar to NBS, Defective DNA NHEJ required for double-strand break repair and V(D)J recombination | ↓ | ↓ | ↓ | – | AR |
| Bloom syndrome | *BLM* | 15q26.1 | Bloom helicase | Chromosomal instability; leukemia; lymphoma; short stature; bird-like face; sun sensitive, hypo-/hyper pigmented, telangiectatic skin; prone to diabetes; lung infections; defect in DNA repair/copy, Ashkenazi Jews | N | N | ↓ | – | AR |

Data abstracted from Webster et al. 1992, Anonymous 2000, Online Mendelian Inheritance in Man 2000, Buckley 2003a, Chapel et al. 2003, Kaneko and Kondo 2004, Notarangelo et al. 2004, Vihinen 2004, Bonilla and Geha 2006. Abbreviations: AD, Autosomal dominant; AR, Autosomal recessive; XL–X, linked; M, monocyte/macrophage; L, lymphocyte; NK, Natural killer; ↓, decreased; ↓↓, profoundly decreased; ↑, increased; N, normal; for Serum Ig Column, "All" refers to all isotypes.

differentiation in the thymus due to the absence of IL-2Rα on thymic epithelial cells (Vihinen et al. 2001, Vihinen 2004; Table III).

*CD45 deficiency*

CD45 is a tyrosine phosphatase found on hematopoietic cells that regulates kinases vital for signal transmission through B and T cell antigen receptors. Two cases have been reported (Kung et al. 2000, Tchilian et al. 2001, Buckley 2003a; Table III).

*Winged helix nude deficiency (WHN deficiency)*

Only 2 cases of this $T^-B^+$ SCID have been reported (Vihinen 2004, Auricchio et al. 2005). Notable features are deficiency of mature T cells (decreased CD4 cells and relatively normal CD8 cells), alopecia, and nail dystrophy (Vihinen 2004). The WHN protein is thought to be a transcriptional regulator in the thymus that is involved in T cell development (Table III).

*Immunodeficiency with thymoma*

Another $T^-B^+$ SCID of unknown molecular etiology is the group of disorders known as immunodeficiency with thymoma. This is a PID of adults with panhypogammaglobulinemia, cellular immunodeficiency, and usually benign thymoma. Although the fraction of circulating Ig-bearing B cells is usually normal, Ig production is defective (Litwin 1979, Buckley 2003a). Various cytopenias are sometimes seen (Litwin 1979, Buckley 2003a) (Table III).

### Severe combined immunodeficiency diseases (SCIDs): $T^-B^-$ SCID

*Adenosine deaminase deficiency (ADA deficiency)*

ADA deficiency is the most common autosomal recessive SCID, making up 15% of SCID cases (Bonilla and Geha 2003). These patients are similar to γc SCID patients but may be distinguished by ribcage anomalies and osseochondral dysplasia at the costochondral junctions, iliac joints, and vertebral bodies (Buckley 2003a). ADA deficiency typically causes more severe lymphopenia (absolute counts less than $500/mm^3$) than other SCIDs (Buckley 2003a). NK cell number and function is intact, and if cellular immunity is reconstituted with BMT, B cell function returns (Resta and Thompson 1997). Lack of ADA enzymatic activity allows toxic adenosine metabolites to accumulate. These metabolites result in thymocyte and circulating T and B cell apoptosis (Resta and Thompson 1997). Milder forms with delayed onset and diagnosis have been reported. Although polyethylene–glycol-modified bovine ADA (PEG–ADA) can help by replacing the missing enzyme, the response is not as effective as that from BMT (Zegers and Stoop 1983, Buckley 2003a). Gene therapy has also has resulted in immune reconstitution but some leukemic complications have been reported (Chinen and Puck 2004, Fischer et al. 2004, Ferguson et al. 2005; Table III).

*Recombinase activating gene 1 deficiency (RAG1 deficiency) and recombinase activating gene 2 deficiency (RAG2 deficiency)*

*RAG1* and *RAG2* encode for proteins that control somatic recombination of the T and B cell receptor genes. Without this regulation of the gene recombination, there is no assembly of the receptor genes, no receptors are formed, and T and B cell development is arrested at immature stages (Corneo et al. 2001). These patients have functional NK cells as the predominant cell type in their circulation (Buckley 2003a; Table III). Defects in RAG 1 or 2 are the usual cause of Omenn syndrome.

*Artemis gene product deficiency (Athabascan SCID)*

After RAG1 or 2 make cuts in DNA, the protein produced from the Artemis gene is responsible for repairing the DNA. Artemis gene product deficiency results in a $T^-B^-NK^+$ SCID similar to RAG1 or 2 deficiency (Li et al. 2002). A hallmark of this SCID is increased radiation sensitivity (Table III). Defects in the Artemis gene have also been associated with Omenn syndrome (Bonilla and Geha 2006).

*Reticular dysgenesis*

Also known as SCID with leukopenia, reticular dygenesis is a $T^-B^-$ SCID of unknown molecular etiology. It is characterized by profoundly decreased numbers of B and T cells, hypogammaglobulinemia as well as granulocytopenia and thrombocytopenia (Roper et al. 1985). Deafness is also seen. Only about 30 cases have been reported, a few of which have displayed normal-appearing granulocytes in the blood and one with a normal T cell fraction in cord blood. These facts suggest that the suspected stem cell maturation defect responsible for defective development of T cells, B cells, and granulocytes, may not be complete (Buckley 2003a; Table III).

### Other rare SCIDs

*SCID with cytokine production defects*

Very rare patients with SCID of uncertain molecular etiology have been found that do not produce a single or multiple cytokines. Selective inability to produce IL-2 despite presence of the IL-2 gene has been seen in 2 cases (Litwin 1979, DiSanto et al. 1990). One female with defective ability to transcribe genes for IL-2, IL-3, IL-4, and IL-5 has been reported; the latter deficiency may be due to abnormal binding of nuclear factor of activated T cells (NFAT) to lymphokine gene enhancers (Castigli et al. 1993). Two cases of boys with SCID who had normal appearing circulating lymphocytes but whose T cells could not make Il-2, IFN-$\gamma$, IL-4, or TNF-$\alpha$ were found to have very low levels of NFAT binding to DNA promoter regions of the IL-2 gene (Feske et al. 1996; Table III).

### Combined immunodeficiency diseases (CIDs)

### *Hyper IgM syndromes related to CD40 ligand/CD40 axis: X-linked hyper IgM syndrome (CD40 ligand deficiency) and CD40 deficiency hyper IgM syndrome (CD40 deficiency)*

As opposed to the isolated humoral deficiency in hyper IgM syndrome due to UNG or AICD deficiency, defects in the CD40 ligand/CD40 interaction result in combined immunodeficiency. X-linked hyper IgM syndrome is due to mutations in the gene coding for CD40 ligand (DiSanto et al. 1993). CD40 ligand, a protein on T helper cells, normally interacts with CD40 protein on B cells. This interaction is needed for proper isotype switching, without which the B cells produce only IgM (Seyama et al. 1998). Also, if CD40 is not stimulated, these B cells do not upregulate the costimulatory molecules CD 80/86, which in turn allows T cells to become "tolerogenic". Tolerogenic T cells are thought to cause the increase in malignancy, especially hepatoma, in this disease (Buckley 2003a). The mutation also leads to autoimmune cytopenias. Neutropenia and the intrinsic T cell abnormality is felt to contribute to opportunistic infections (Buckley 2003a). These patients develop the typical infectious complications of combined PIDs and have little lymphoid tissue. The treatment of choice, given the poor prognosis, is BMT, but IVIG is used as well (Buckley 2003a). Prophylaxis against *Pneumocystis* pneumonia is also routinely given (Bonilla and Geha 2003). CD40 deficiency presents similarly to CD40 ligand deficiency and is treated in the same fashion (Table III).

### *Purine nucleoside phosphorylase deficiency (PNP deficiency)*

PNP deficiency is another disorder of purine metabolism like ADA deficiency. T cells are low in number but not absent (Buckley 2003a). B cells are normal in number and serum Igs are usually normal. NK cells are increased. The defect is due to accumulation of toxic metabolites due to the absence of the PNP enzyme. Many patients have neurologic symptoms and autoimmunity. Without BMT, the disease is fatal in childhood (Myers et al. 2004, Notarangelo et al. 2004) (Table III).

### *Omenn syndrome*

Omenn syndrome is a CID characterized by hepatosplenomegaly, hypereosinophilia, erythroderma, desquamation, increased serum IgE, and diarrhea in newborns (Aleman et al. 2001). This syndrome is due to partial deficiency in RAG1 or RAG2, with the signs mediated by oligoclonal, activated T cells in the circulation. B cells are usually reduced in the blood (Villa et al. 1999) (See RAG deficiency above and Table III below).

### *MHC class I deficiencies: Transporter associated protein 1 deficiency (TAP-1 deficiency); transporter associated protein 2 deficiency (TAP-2 deficiency); and TAP-binding protein (tapasin) deficiency (tapasin deficiency) MHC class II deficiencies: CIITA; RFX5; RFXAP; and RFXANK*

The MHC antigens (MHC class I or MHC class II) bound to processed antigen are recognized by the T cell receptor, allowing activation of T cells. MHC I is found on all nucleated cells and platelets and recognized by CD8$^{+}$T cells, while MHC II, found on B cells, monocyte-macrophages, antigen-presenting cells, and some T cells, is recognized by CD4$^{+}$T cells.

*Transporter-associated protein 1 (TAP-1)* and *transporter-associated protein 2 (TAP-2)* are two genes encoding proteins that normally transport processed antigen to the MHC I molecule. Mutations in either TAP-1 or TAP-2 result in destruction of the MHC I proteins before they appear on the cell surface, leading to a combined immunodeficiency with decreased CD8$^{+}$T cells but normal numbers of CD4$^{+}$T cells (de la Salle et al. 1994, Gadola et al. 2000). However, MHC I is present in normal amounts in serum (Buckley 2003a). Peripheral B cells and Ig levels are normal. This immunodeficiency is milder than SCID and often presents at a later age. Vasculitis is common. A similar deficiency has been reported due to mutations of genes coding for a protein coined tapasin, which acts as a molecular chaperone for TAP (Yabe et al. 2002).

MHC II defects result in a more severe immunodeficiency than that of MHC I defects, but still milder than that of SCID. This is usually seen in patients of North African ancestry and presents with very low CD4$^{+}$ counts and normal CD8$^{+}$ counts (Buckley 2003a). These patients have abnormal T cell and subsequent antibody responses to specific antigens and underdeveloped thymus and lymphoid tissue. MHC II defects have been reported that are due to mutations in genes that code for various components (RFX5, RFXAP, and RFXANK) of a multiprotein complex called RFX, which binds a promoter on the MHC II gene (Steimle et al. 1995, Villard et al. 1997). The same immunodeficiency may result from a mutation in the gene coding for MHC Class II transactivator (CIITA), a protein that controls inducibility and cell-specific expression of MHC II (Zhou and Glimcher 1995, Buckley 2003a; Table III).

*Other T cell activation defects: Zeta-associated protein 70 (ZAP-70); p56 Lck; CD3$\gamma$; and CD3$\epsilon$*

Normal T cell signal transduction involves recognition of the antigen–MHC complex by the TCR, as noted above in the MHC deficiency section. Subsequently, this signal is transduced into the cytoplasm via the CD3 complex, which activates various protein tyrosine kinases such as ZAP-70, p56 Lck, Fyn, and Syk (Buckley 2003a). These kinases phosphorylate phospholipase C and activate other proteins, all of which results in distal signaling events such as activation of protein kinase C and calcium influx, which lead to transcription of cytokine genes such as IL-2. Together these events result in T cell activation. If any of the components of this complex cascade are defective or absent, immunodeficiency can result.

Defects in the gene encoding ZAP-70 result in a CID with CD8 lymphopenia with normal numbers of abnormally functional CD4 cells, normal NK cell function, and variable serum Ig levels (Chan et al. 1994, Elder et al. 1994).

p56 Lck gene mutation has been described in an infant presenting with CD4 lymphopenia, low serum Igs, but normal B and NK cell numbers (Timon et al. 1993, Buckley 2003a).

Unlike defects in CD3$\delta$, which results in SCID, mutations in CD3$\gamma$ or CD3$\epsilon$ result in a combined immunodeficiency with normal numbers of circulating T cells (Timon et al. 1993, Buckley 2003a). However, the T cells have abnormal proliferative responses, and clinical problems have included severe viral and bacterial infections and autoimmune phenomena (Table III).

*CD8 deficiency*

A complete absence of CD8 cells has been reported in one patient found to have mutations in part of the gene coding for CD8 called *CD8a* with normal Ig levels, NK cell function, but recurrent infections (de la Calle-Martin et al. 2001; Table III).

*DNA repair defects: Ataxia–telangiectasia (AT); ataxia–telangiectasia-like syndrome; Nijmegan breakage syndrome; DNA ligase IV deficiency; DNA ligase I deficiency; and Bloom syndrome*

Various defects in DNA repair can lead to PID. A common feature is increased sensitivity to ionizing radiation. AT is a syndrome with immunodeficiency, cerebellar ataxia, oculocutaneous telangiectasia, sensitivity to ionizing radiation, and frequent malignancy (lymphoreticular, leukemia, and others) (Bonilla and Geha 2003). The disorder is due to mutation in the *AT-mutated (ATM)* gene, whose product normally detects damaged DNA and prevents the damaged cell from dividing (Perlman et al. 2003). The mutant ATM allows such damaged cells to divide, which leads to chromosomal instability and increases risk of malignancy. The mutation also interferes with lymphocyte development, resulting in decreased circulating T cells and variable decrease in antibodies, presumably due to interference with TCR and B cell receptor assembly (Bonilla and Geha 2003). Since $\alpha$-fetoprotein is elevated in 95% of patients, this is useful diagnostically (Bonilla and Geha 2003). Unfortunately, AT is treated only with supportive care; toxicity of pre-BMT myeloablation is severe and does not allow successful BMT (Bonilla and Geha 2003). Several other PIDs due to defective DNA repair are presented in Table III.

### Defects of innate immunity

*Defective regulation of the nuclear factor of $\kappa$B (NF-$\kappa$B) pathway: Nf-$\kappa$B essential modulator (NEMO) defect and inhibitor of $\kappa$B (I$\kappa$B) defect*

Nf-$\kappa$B is a transcription factor that controls production of molecules that play important roles in the processes of a normal immune and/or inflammatory response; the downstream molecules include IL-1, IL-2, Il-6, IL-8, G and GM-CSF, and TNF (Bonilla and Geha 2003). Normally, an inhibitor of Nf-$\kappa$B (called I$\kappa$B) is prevented from inactivating Nf-$\kappa$B because another molecule, I$\kappa$B kinase (IKK) phosphorylates I$\kappa$B, degrading this inhibitor, and allowing Nf-$\kappa$B to remain active. Partial-loss-of function mutations in the $\gamma$ chain of I$\kappa$B kinase (also known as NEMO, for Nf-$\kappa$B essential modulator) means that I$\kappa$B is not degraded and therefore inhibits Nf-$\kappa$B activity (Orange et al. 2004a, Bonilla and Geha 2006). This disrupts immune function, resulting in the NEMO syndrome, which is characterized by specific antibody deficiency to polysaccharides, infections (especially mycobacterial), and almost always anhidrotic ectodermal dysplasia. This NEMO syndrome is also known as EDA-ID (for ectodermal dysplasia associated immunodeficiency). Some patients with this NEMO mutation have had hyper IgM, and two patients reported do not have ectodermal dysplasia, highlighting the variable nature of the NEMO syndrome (Niehues et al. 2004, Orange et al. 2004b). Another NEMO mutation causes a syndrome with additional features such as osteopetrosis and lymphedema and is abbreviated OL-EDA-ID. A mutation that activates I$\kappa$B can cause the EDA-ID

phenotype, since an activated IκB allows IκB to inhibit Nf-κB (Courtois et al. 2003; Table IV). Additional defects of innate immunity including those listed below are described in Table IV. Defects in complement may also be considered defects of innate immunity.

*IL-1 receptor-associated kinase 4 deficiency (IRAK-4 deficiency)*

Most of the toll-like receptors (TLRs) use a signaling pathway that involves a kinase called IRAK-4 (Notarangelo et al. 2004). IRAK-4 is upstream of NEMO in the NFκB signaling pathway described above (Vihinen 2004). The defect in IRAK-4 affects lymphocyte and monocyte inflammatory responses to pathogens and results in pyogenic bacterial infections (Notarangelo et al. 2004).

*Warts, hypogammaglobulinemia, infections, and myelokathexis syndrome (WHIM syndrome)*

Another defect of innate immunity is the WHIM syndrome (Stiehm 1993). Treatment-resistant warts due to HPV infection, hypogammaglobulinemia with associated bacterial infections that tend to be mild, and myelokathexis (retention of mature myeloid cells in the marrow) are the hallmarks of this disease (Tarzi et al. 2005). It is the first described PID due to abnormality of a chemokine receptor. In this disorder, mutation in the gene coding for a leukocyte chemokine receptor called CXCR4 results in increased response of CXCR4 to its ligand CXCL12 (Notarangelo et al. 2004). This interaction is responsible for homing of neutrophils and other hematopoietic cells to the bone marrow and proper release of mature leukocytes from the marrow (Vihinen 2004). The mutation results in retention of mature neutrophils in the marrow and neutropenia. Peripheral lymphocyte numbers and function are variably deficient (Vihinen 2004). Infections often respond to antibiotics, aided by the inflammation-induced release of neutrophils from the bone marrow despite the defect. G-CSF, GM-CSF, and IVIG are also used in managing WHIM syndrome (Vihinen 2004).

### Phagocyte defect-associated PIDs

*Chronic granulomatous disease (CGD): X-linked CGD (XCGD); p22phox deficiency (p22phox CGD); p47phox deficiency (p47phox CGD); p67phox deficiency (p67phox CGD)*

Phagocytic disorders typically result in poor wound healing and susceptibility to bacterial (particularly catalase-positive), mycobacterial, and fungal infections (Figure 1). CGD is the prototypical disorder of phagocyte function that presents in children and occasionally in adults. Both X-linked and autosomal recessive forms are characterized by infections due to isolated inability to effectively destroy intracellular pathogens and, as a result of this ineffective killing, granuloma formation in any organ. Initially, infections are from catalase-positive bacteria (*Staphylococcus aureus*, *Pseudomonas*, *Salmonella*, and *Serratia marcescens*), however, not from catalase-negative pathogens (*Hemophilus influenzae* or *Streptococcus pneumoniae*). Abscesses and osteomyelitis are frequent complications. Aspergillus and other fungal disease is prominent.

The fundamental defect underlying all of the CGDs is a mutation in any of the closely related proteins making up the phagocyte oxidase complex (also known as nicotinamide adenine dinucleotide phosphate oxidase or NADPH oxidase), which normally allows the cell to form hydrogen peroxide and superoxide radicals that can directly kill phagocytosed pathogens during a respiratory burst (Bonilla and Geha 2003). The NADPH oxidase complex is made of two membrane protein subunits (gp91phox and p22phox) and two intracytoplasmic proteins (p67phox, p47phox, and p40phox). The NADPH oxidase complex has its activity and assembly regulated by two intracytoplasmic membrane-associated GTPase proteins (Rac2 and Rap1). Mutation in the gene coding for gp91phox is the most common cause of CGD, and is the defect in XCGD, while less common defects in the *p22phox*, *p47phox*, or *p67phox* genes have been shown to cause the autosomal recessive forms of CGD. Because Rac2 also is involved in regulation of the actin cytoskeleton, Rac2 gene mutations result in leukocyte adhesion deficiency (LAD) with associated oxidative killing defect (Notarangelo et al. 2004). Although not yet reported, CGD due to defects in p40phox or Rap1 is also possible.

Diagnosis of CGD is established by documenting the inability of patients' neutrophils to generate superoxide after they are stimulated with phorbol myristate acetate (PMA) or after they phagocytose (Buckley 2003a). Superoxide generation can be detected by measuring chemiluminescence using the nitroblue tetrazolium (NBT) dye reduction test or by using a flow cytometry-based respiratory burst assay (Buckley 2003a). Both are abnormal in CGD.

The frequent use of antibiotics has reduced childhood death due to CGD, and has made aspergillus infection a more frequent cause of fatality in CGD (Czarnetzki 1989, Buckley 2003a). Aspergillus infections may be treated with amphotericin B, regular normal granulocyte infusions, and maintenance oral antifungals. IFN-γ injected subcutaneously seems to reduce serious infections without improving phagocyte killing abilities (Mamishi et al. 2005). BMT has not been very successful, unless done very early, due to

Table IV. Defects of innate immunity.

| Disorder | Abnormal gene | Abnormal genetic locus | Abnormal gene product | Classic/associated features | Affected cells | Functional defect | Inheritance |
|---|---|---|---|---|---|---|---|
| Ectodermal dysplasia associated immunodeficiency (EDA-ID) | | | | | | | |
| Nf-κB essential modulator (NEMO) defect | *IKBKG* | Xq28 | NEMO (γ chain of IκBK) | Anhidrotic ectodermal dysplasia (usually), lack of antibody response to polysaccharides, various infections (Mycobacteria, bacterial), possible Hyper IgM | L + M | NFκB pathway | XL |
| Inhibitor of κB (IκB) Defect | *IKBA* | ? | α chain of IκB | Anhidrotic ectodermal dysplasia, T-cell defect, infections | L + M | NFκB pathway | AD |
| Defects of toll-like receptor signalling | | | | | | | |
| Il-1 receptor-associated kinase 4 (IRAK-4) deficiency | *IRAK4* | Chr.4 | IRAK-4 | Bacterial infection, standard screening tests for PID usually normal | L + M | IRAK-4 is critical for signaling through majority of TLRs | AR |
| Chemokine receptor defect | | | | | | | |
| Warts, hypogammaglobulinemia, infections, and myelokathexis (WHIM) syndrome | *CXCR4* | 2q21 | CXCR4 | Hypogammaglobulinemia, low B cell numbers, severe neutropenia, warts (HPV infection), retention of mature PMNs in bone marrow (myelokathexis) | G + ?L | Increased response of CXCR4 chemokine receptor to its ligand CXCL12 (interaction homes PMN to marrow so they stay there | AD |

Data abstracted from Anonymous 2000, Online Mendelian Inheritance in Man 2000, Buckley 2003a, Chapel et al. 2003, Notarangelo et al. 2004, Vihinen 2004, Bonilla and Geha 2006. Abbreviations: AD, Autosomal dominant; AR, Autosomal recessive; XL, X-linked; M, monocyte/macrophage; L, lymphocyte; NK, Natural killer; ↓, decreased; ↓↓, profoundly decreased; ↑, increased; N, normal; for Serum Ig Column, "All" refers to all isotypes.

difficulty in managing chronic infections during transplant-related marrow ablation of the host (Czarnetzki 1989, Buckley 2003a; Table V).

*Leukocyte adhesion deficiency (LAD): LAD type 1 (LAD1); LAD type 2 (LAD2); LAD type 3 (LAD3); and LAD with Rac2 deficiency (Rac2 LAD)*

Defects in proteins involved in leukocyte rolling, adhesion, and cytoskeletal regulation make up the group of phagocyte immunodeficiencies called LAD. All four types are characterized by poor wound healing, skin ulcers, gingivitis/periodontitis, delayed separation of the umbilical cord, leukocytosis, and bacterial and fungal infections.

LAD1 is due to mutation of the gene encoding CD18, the common chain of integrin heterodimers (Bonilla and Geha 2003). Without CD18, the integrins, including LFA-1 (CD11a/CD18), Mac-1 (also known as CR3) (CD11b/CD18), and CR4 (CD11c/CD18), are not formed. Leukocyte function associated molecule 1 (LFA-1) on leukocytes binds adhesion molecules, such as intercellular adhesion molecule 1 (ICAM-1), on endothelial cells. This interaction is vital for adhesion before diapedesis of leukocytes such as neutrophils (Bonilla and Geha 2003). Without LFA-1, leukocytes stay marginated in the blood vessels and are unable to reach sites of infection. LAD-1 patients may therefore have extremely high blood leukocyte counts. BMT may be curative but routine antibiotics and sometimes granulocyte transfusion are used (Vihinen 2004).

LAD2 is due to a defective GDP-fucose transporter, resulting in inability to fucosylate glycoproteins, including sialyl–Lewis–X, the ligand for E and P selectins (Vihinen 2004). The interaction between fucosylated sialyl–Lewis–X on leukocytes and selectins on endothelial cells is needed for the initial binding of the leukocyte to the vessel wall (Bonilla and Geha 2003). This defect results in a clinical picture similar to LAD1, but additionally, patients with LAD2 defects have the Bombay (hh) blood group, mental retardation, and dysmorphic features. Oral fucose can reduce fevers and infections (Vihinen 2004).

A less well-characterized LAD is LAD3. Although a specific genetic locus or mutation is not confirmed, the defect is felt to be due to abnormality of Rap1, a regulatory GTPase that is thought to be involved in activation of integrins (and that has a regulatory role in the NADPH oxidase). Abnormality of the former function impairs leukocyte adhesion (Notarangelo et al. 2004). LAD3 results in a clinical picture similar to LAD1 with an additional bleeding propensity (Notarangelo et al. 2004).

LAD with Rac2 deficiency results in a clinical picture similar to LAD due to abnormal leukocyte adherence, chemotaxis, and degranulation (Notarangelo et al. 2004). Rac2, a GTPase involved in the regulation of the NADPH oxidase, also regulates the actin cytoskeleton. Abnormalities of this cytoskeletal regulation lead to these leukocyte adhesion and degranulation problems. The defective regulation of the NAPDH oxidase due to Rac2 deficiency leads to defective superoxide radical formation, as seen in CGD due to Rac2 deficiency (Notarangelo et al. 2004).

*Chediak–Higashi syndrome*

Chediak–Higashi syndrome is a disorder characterized by partial oculocutaneous albinism, variable neurological deficits, and severe bacterial infections (especially by *Staphylococcus aureus*) (Vihinen 2004). A fatal "accelerated phase" of lymphoproliferation with hemophagocytosis and encephalopathy is often fatal (Bonilla and Geha 2003). Malignant lymphoma may be seen. The defect is due to mutations in a lysosomal trafficking protein called LYST (Bonilla and Geha 2003). Without this normal protein, any lysosome-forming cells, including neutrophils, are unable to form normal phagolysosomes and melanosomes, leading to the clinical findings (Notarangelo et al. 2004). Abnormal transport to and from the lysosomes is associated with uncontrolled granule fusion, which leads to defective neutrophil granules. The immunophenotype of this disease includes low NK and cytotoxic T cell function with normal blood T and B cell levels and Ig levels. BMT can be curative.

*The severe congenital neutropenias: Kostmann syndrome (congenital neutropenia); and cyclic neutropenia; X-linked neutropenia/myelodysplasia*

Kostmann syndrome (congenital neutropenia) describes a PID with persistent absolute neutropenia ($<500/mm^3$) resulting in frequent bacterial infections, newborn temperature instability, and in one subgroup, myelodysplasia (Cham et al. 2002). Mutations in the gene coding for Elastase 2 (*ELA2*) makes up about two-thirds of cases, with mutations GFI1 making up the remainder (Notarangelo et al. 2004, Vihinen 2004). Abnormal myeloid differentiation with granulocytes arrested at the promyelocytic stage is seen (Notarangelo et al. 2004, Vihinen 2004). *ELA2* mutations are thought to result in mistrafficking of neutrophil elastase, which is important in degrading pathogen virulence factors and in the leukocyte life cycle (Notarangelo et al. 2004, Vihinen 2004). *GFI1* is a proto-oncogene and likely a transcription factor involved in regulating neutrophil cell division and repressing *ELA2* transcription (Notarangelo et al. 2004, Vihinen 2004). Mutations in the granulocyte colony stimulating factor (G-CSF) receptor seen in some cases of Kostmann syndrome are now believed to be acquired defects, possible due to underlying genetic instability (Bonilla and Geha 2006). Recombinant G-CSF (rGCS-F) treatment can reduce infections and improve survival in severe congenital neutropenia (SCN) (Notarangelo et al. 2004, Vihinen 2004).

Cyclic neutropenia is a PID characterized by blood neutrophil, lymphocyte, platelet, monocyte, and reticulocyte levels that cycle between normal and zero with a 21 day periodicity (Notarangelo et al. 2004, Vihinen 2004). It is due to autosomal dominant mutation in *ELA2*, with resulting abnormal elastase trafficking.

In X-linked neutropenia/myelodysplasia, there is loss of autoinhibition of Wiskott–Aldrich syndrome protein (WASP) (Devriendt et al. 2001). WASP is involved in regulation of the actin cytoskeleton of hematopoietic cells, including neutrophils. This constitutively activating mutation in WASP causes neutropenia and myelodysplasia in this disorder (Devriendt et al. 2001). Other mutations in WASP, as described below, result in the Wiskott–Aldrich syndrome and in X-linked thrombocytopenia (Oda and Ochs 2000, Notarangelo et al. 2002).

### Complement deficiency-associated PIDs

There have been reported deficiencies of every soluble complement component except for factor B (Yabe et al. 2002, Bonilla and Geha 2003). These are relatively uncommon PIDs. The complement system plays several vital roles in human immunity, which can be divided into three groups: defense against infectious agents, connection between the innate and adaptive immune systems, and elimination of waste products and dying cells (Walport 2001a,b). Although partial deficiency of a complement component generally does not lead to infectious disease, complete deficiencies result in infectious complications from bacterial pathogens, particularly *Neisseria* (Bonilla and Geha 2003; Figure 1). Defects of the classic and late complement pathways tend to be associated with less severe infectious disease than alternative pathway deficiencies, particularly with initial exposure to the pathogen, when the alternative pathway is most important (Buckley 2003a). Autoimmune disease such as systemic lupus erythematosis is more common in patients with partial and complete complement deficiencies, particularly those with early component defects (Walport 2001a,b). Disruption of the clearance of immune complexes when these deficiencies are present is a proposed reason for increased susceptibility to autoimmunity in these diseases (Buckley 2003a). Defects in some complement components lead to non-infectious disease, as is the case with hereditary angioedema due to C1 esterase inhibitor deficiency. Unfortunately, there is no specific replacement therapy for these deficiencies, and treatment is largely appropriate antibiotic use and fresh–frozen plasma infusion for emergent replacement of some complement components (Buckley 2003b, Vihinen 2004). Autoimmune disease can be treated with immunosuppressive medicines (Bonilla and Geha 2003). The characteristics of immunodeficiency due to the various complement components are included in Table VI.

### Other well-defined immunodeficiency syndromes and PIDS

### *Hyper IgE syndrome (Job's syndrome)*

Hyper IgE syndrome is of unknown etiology and is marked by extremely elevated serum IgE levels, severe and recurrent abscess formation in lungs, skin, joints, and other organs due primarily to *Staphylococcus aureus*, pneumatocele formation, and non-atopic dermatitis (Shemer et al. 2001). Patients have coarse facial features including a prominent forehead, deep-set eyes, a broad nasal bridge, a wide and fleshy nasal tip, and facial asymmetry (Grimbacher et al. 1999, O'Connell et al. 2000, Buckley 2003a, Grimbacher et al. 2005). Many display scoliosis, hyperextensible joints, delayed shedding of the primary teeth, and osteopenia with easily-fractured bones (Grimbacher et al. 1999, O'Connell et al. 2000, Buckley 2003a, Grimbacher et al. 2005). Males and females are both susceptible. Usually hyper IgE syndrome is autosomal dominant, but recently an autosomal recessive form has been described that is characterized by more severe viral infections, central nervous system vasculitis (in some) but a lack of skeletal involvement, dental pathology, or pneumatocele formation (Bonilla and Geha 2006).

Immunologically, in addition to the serum IgE in the tens of thousands, IgD is high, and the other isotypes are normal (Grimbacher et al. 1999, O'Connell et al. 2000, Grimbacher et al. 2005). There is a marked eosinophilia of blood, sputum, and lymphoid and pulmonary tissues. There is a decreased humoral (especially anamnestic) and cellular immune response to new antigens despite normal fractions of CD4 and CD8 subtypes (Buckley 2003a). Lymphocytes proliferate normally when stimulated with mitogens but not antigens; there is decreased CD45RO, or memory phenotype, T cells (Buckley 2003a). Prognosis largely depends on the rapidity of initiation of aggressive and long-term anti-staphylococcal (and other pathogen-specific antibiotics) treatments (Buckley 2003a,b). If this is done, prognosis is good, but without it, patients develop progressive pulmonary disease. Surgical correction of persistent or infected pneumatoceles is also standard of care (Table VII).

*Wiskott–Aldrich syndrome (WAS)*

WAS, also classified as a combined PID, is marked by thrombocytopenic purpura, small defective platelets, eczema, and infection with encapsulated bacteria at younger ages followed by opportunistic pathogens later (Buckley 2003a). The typical presentation occurs during infancy with bleeding problems, followed by eczema, infections, and often other atopic disease (Buckley 2003a). Cancers, especially lymphoreticular malignancy, bleeding, and infections are the usual reasons for death, which occurs before the teen years (Paller 1995, Oda and Ochs 2000). Autoimmunity in the form of cytopenias and vasculitis is frequent (Buckley 2003a). Synthesis and catabolism of Igs are increased, leading to variable levels, but the classic pattern shows elevated IgA and IgE with low IgM and normal or mildly low IgG. The antibody response is blunted, particularly to polysaccharides, but later also to proteins. Absence of isohemagglutinins due to this poor polysaccharide-induced antibody response may aid in diagnosis (Buckley 2003a) The cellular immune system is also defective, with reduced numbers of T cells with poor response to mitogens (Buckley 2003a). Finding non-random X chromosome lyonization in several hematopoietic cell lines or detecting the abnormal gene can identify carriers, while prenatal diagnosis is possible via chorionic villus or amniotic fluid sampling (Marone et al. 1986, Paller 1995, Oda and Ochs 2000).

The molecular defect results from various mutations in the gene coding for WAS protein (WASP). WASP, which is found only in lymphocytes and megakaryocytes, is critical for actin polymerization, and therefore, normal cytoskeletal structure and function (Oda and Ochs 2000). Other mutations in the WASP gene can result in isolated X-linked thrombocytopenia or X-linked neutropenia (Devriendt et al. 2001, Notarangelo et al. 2002).

HLA-identical BMT can cure WAS, but haploidentical BMT has been less successful (Conley et al. 2003). Problematic thrombocytopenia is treated with splenectomy. IVIG therapy is also routinely used (Table VII).

*DiGeorge syndrome (DGS)*

DGS is also known as thymic hypoplasia and sometimes grouped with other combined PIDs. It is along a spectrum of disease, also containing the velocardiofacial syndrome, usually due to deletions on chromosome 22q11.2 (the DiGeorge chromosomal region) (Bonilla and Geha 2006). The deletion results in abnormal development of the third and fourth pharyngeal pouches during human embryonic development. Numerous structures that develop from these pouches, including the thymus and parathyroid glands, are underdeveloped or absent in DGS. Abnormalities of the heart (atrial septal defects, conotruncal disorders), great vessels (right-sided aortic arch), abnormal facies (wide-set eyes, low-set and notched ears, mandibular hypoplasia, short upper lip philtrum), and esophageal atresia are seen in DGS since these structures develop from similar areas and at the same time as the thymus and parathyroids (Sullivan 2004). Newborns may have hypocalcemic seizures due to hypoparathyroidism (Buckley 2003a). Clinically, patients with milder thymic and parathyroid hypoplasia have few serious infections and do not have failure to thrive. This is called partial DGS, while those with more significant thymic hypoplasia or aplasia of the thymus and the resulting SCID-like immunodeficiency pattern that results, are said to have complete DGS (Sullivan 2004).

Immunologically, DGS usually has mild lymphopenia, with a variable decrease in T cells with reduced response to mitogens depending on the degree of thymic hypoplasia. The T cells present seem to be intrinsically normal in many patients, but some with complete DGS may have populations of abnormally functioning T cells (Buckley 2003a, Bonilla and Geha 2006). The thymus-dependent lymphoid tissues are similarly variably hypoplastic.

Although most cases are associated with microdeletions in 22q11.2, others include deletions on chromosome 10p13, and more recently, mutations in a transcription factor called T-box-1 (Kim and Basson 2001, Sullivan 2004).

Treatment of DGS depends on the severity. Partial DGS requires no treatment, while complete DGS is fatal if not immunologically reconstituted via BMT or, according to some experts, thymic epithelial explants (Sullivan 2004). Cardiac lesions need to be treated surgically (Table VII).

*X-linked lymphoproliferative syndrome (XLP)*

XLP is an excellent example of a genetic disorder whose expression seems to require the correct environmental stimulus to become active. Boys with XLP typically are seemingly healthy until they have infectious mononucleosis due to Epstein–Barr virus (EBV). In response to the EBV infection, the XLP patient will have fulminant infectious mononucleosis, often with hepatic necrosis and death (50%), hypogammaglobulinemia (30%), or B cell lymphoma (20%) (Morra et al. 2001). Patients surviving the EBV infection typically develop hypogammaglobulinemia or lymphoma later (Buckley 2003a).

A protein called signalling lymphocyte activation protein (SLAM) is found on T cells, thymocytes, and NK cells. In patients without XLP, a protein called SLAM-associated protein (SAP) inhibits signal transduction via SLAM and thus prevents T cell proliferation in response to EBV. SAP also interacts with NK cells. The gene coding for SAP, *SH2D1A*, is

Table V. Phagocyte abnormalities.

| Disorder | Abnormal gene | Abnormal genetic locus | Abnormal gene product | Classic/associated features | Affected cells | Functional defect | Inheritance |
|---|---|---|---|---|---|---|---|
| Chronic granulomatous disease (CGD) | | | | | | | |
| X-linked CGD (gp91phox CGD) | *CYBB* | Xp21.1 | gp91phox | Poor wound healing, granulomas, catalase- + bacterial and fungal infections, gingivitis, delayed separation of the cord, osteomyelitis | P + M | Oxidative burst-dependent killing impaired due to defective NADPH oxidase components | XL AR AR |
| p22phox deficiency (p22phox CGD) | *CYBA* | 16q24 | p22phox | | | | AR |
| p47phox deficiency (p47phox CGD) | *NCF1* | 7q11.23 | p47phox | | | | |
| p67phox deficiency (p67phox CGD) | *NCF2* | 1q25 | p67phox | | | | |
| Leukocyte adhesion deficiency (LAD) | | | | | | | |
| LAD type 1 (LAD1) | *ITGB2* | 21q22.3 | CD18 (common chain of β2 integrins) | Poor wound healing, skin ulcers, gingivitis/periodontitis, delayed separation of the umbilical cord, leukocytosis, bacterial and fungal infections, BMT may be curative but routine antibiotics and sometimes granulocyte transfusion are used | P + M + L + NK | CD18 defect results in defective β2 integrins (α/CD18 heterodimers), needed for leukocyte adhesion to EC adhesion molecules such as ICAM-1 | AR |
| LAD type 2 (LAD2) | *SLC35C1* | 11p11.2 | FUCT1 GDP-fucose transporter | Like LAD 1 plus Bombay (hh) blood group and mental retardation, dysmorphic features, oral fucose can reduce fevers and infections | P + M | Mutations in the guanosine diphosphate-fucose transporter gene results in inability to fucosylate glycoproteins, including sialyl-Lewis-X, the ligand for E and P Selectins (needed for leukocyte rolling chemotaxis) | AR |
| LAD type 3 (LAD3) | ? | ? | ?Rap 1 | Like LAD 1 plus bleeding propensity | P + M + L + NK | Leukocyte adherence is abnormal due to defective activation of integrins | AR |
| LAD with Rac2 deficiency (Rac2 LAD) | *RAC2* | 22q12.3–q13.2 | RAC2 | Like LAD 1, BMT can be curative | P | Abnormal P chemotaxis, adherence, and respiratory burst. Rac2, part of the NADPH oxidase complex, helps regulate the actin cytoskeleton (needed for chemotaxis and degranulation) and superoxide production via the NADPH oxidase | AD |
| Neutropenic disorders | | | | | | | |
| Kostmann syndrome (congenital neutropenia) | *ELA2** *2/3 of cases | GFI1 1p35–p34.3 | Elastase 2* GFI1 *2/3 of cases | Persistent absolute neutropenia $<500/mm^3$; frequent bacterial infections; newborn temperature instability irritability; one subgroup with myelodysplasia; rG-CSF treatment can reduce infections and improve survival | P | Abnormal myeloid differentiation with granulocytes arrested at promyelocytic stage; ELA2 mutations thought to result in mistrafficking of N elastase (important in degrading pathogen virulence factors and leukocyte life cycle):GFI1 defects may repress elastase | AD |

Table V – *continued*

| Disorder | Abnormal gene | Abnormal genetic locus | Abnormal gene product | Classic/associated features | Affected cells | Functional defect | Inheritance |
|---|---|---|---|---|---|---|---|
| Cyclic neutropenia | *ELA2* | 19p13.3 | Elastase 2 | Neutrophils, lymphocytes, platelets, monocytes, and reticulocytes levels in blood cycle between normal zero with a 21 day periodicity; rG-CSF treatment can reduce infections and improve survival | P | ELA2 mutations thought to result in mistrafficking of N elastase (important in degrading pathogen virulence factors and leukocyte life cycle) | AD |
| X-linked neutropenia/ myelodysplasia | *WAS* | Xp11.4–p11.21 | WAS protein (WASP) | Neutropenia; other mutations in WASP lead to WAS and X-linked thrombocytopenia | P + M | Loss of autoinhibition of WASP impairs cytoskeletal control in hematopoietic cells, leading to the neutropenia | XL |
| Other phagocyte defects | | | | | | | |
| Chediak–Higashi syndrome (CHS) | *CHS1* | 1q42.1–q42.2 | LYST | Partial oculocutaneous albinism; severe bacterial infections; giant lysosomes; low NK and CTL function; Normal blood T/B cell levels and Ig; accelerated encephalopathic stage; malignant lymphoma; BMT may cure | P + all lysosome containing cells | Mutation in LYST (lysosomal trafficking regulator) impairs intracellular transport to and from the lysosome, causing giant inclusion bodies in a variety of cell types, uncontrolled granule fusion leading to defective granules in neutrophils, and thus failed chemotaxis. | AR |
| Griscelli syndrome, type 1 (GS1) | *MYO5A* | 15q21 | Myosin 5A | Partial pigmentary dilution or albinism with silvery gray hair, frequent infections, stable neurologic abnormalities; never have hemophagocytic syndrome; BMT may help; [GS, type 3 (isolated hypopigmentation phenotype) is also due to a MYO5A defect)] | No abnormality observed | Myosin 5A is a motor that may be involved in melanosome transport, or may be required for cell polarization needed for dendrite formation | AR |
| Griscelli syndrome, type 2 (GS2) | *RAB27A* | 15q21 | RAB27A protein | Partial albinism (silvery gray hair); low NK and CTL function; normal blood T/B cell levels and Ig; anemia, neutropenia; giant lysosomes of CHS absent;, frequent infections; accelerated phase of the disease with fever, jaundice, hepatosplenomegaly, lymphadenopathy, pancytopenia and generalized lymphohistiocytic infiltrates of various organs including the central nervous system | P + T + M | RABD27 protein is key effector of intracellular vesicular transport, so regulates cytotoxic granule exocytosis and affects T cell and macrophage activation; if it occurs, uncontrolled T cell and macrophage activation (Hemophagocytic syndrome) is fatal without BMT | AR |
| β-actin deficiency | *ACTB* | 7p15–p12 | Cytoplasmic β-actin | Mental retardation; short stature | P + M | Mutation in cytoskeletal protein impairs leukocyte motility | AD |

Table V – *continued*

| Disorder | Abnormal gene | Abnormal genetic locus | Abnormal gene product | Classic/associated features | Affected cells | Functional defect | Inheritance |
|---|---|---|---|---|---|---|---|
| Neutrophil-specific granule deficiency | *CEBPE* | 14q11.2 | C/EBPE (Ccaat/ enhancer-binding protein ϵ) | Severe bacterial infections; P with bilobed nuclei; P with abnormal nuclear morphology, lacking primary, specific, and tertiary granule proteins; rG-CSF treatment can reduce infections and improve survival; BMT is an option | P | Defective transcription factor (C/EBPE) leads to lack of specific or secondary granules in mature neutrophils and eosinophils with defective chemotaxis and killing | AR |
| Myeloperoxidase deficiency | *MPO* | 17q23.1 | MPO (myelo-per-oxidase) | Most people with defect are asymptomatic; common (1 in 2000); if immunodeficient, have defective candidal killing and increased malignancy; may have eosinophilia; visceral infections often seen with DM | P + M | Defect in P and M MPO, which is involved in oxidative killing of pathogens | AR |
| Glucose 6-phosphate dehydrogenase deficiency | *G6PD* | Xq28 | G6PD | Most asymptomatic but some with neonatal jaundice, infections, drug-induced hemolytic anemia | P + M | Defective G6PD, which is needed to produce NADPH as reduction agent for oxidative stresses; defect leads to faulty superoxide production and killing | XL |
| Shwachman–Bodian–Diamond syndrome (SBDS) | *SBDS* | 7q11 | SBDS protein | Pancytopenia (may have malignant transformation); exocrine pancreatic insufficiency; chondrodysplasia; bone defects (metaphyseal dysostosis, pectus carinatum), cutaneous defects (ichthyosis), psychomotor retardation; infections; FTT | P | Defective chemotaxis; SBDS protein may be involved in RNA metabolism | AR |

Data abstracted from Anonymous 2000, Devriendt et al. 2001, Buckley 2003a, Chapel et al. 2003, Notarangelo et al. 2004, Vihinen 2004, Bonilla and Geha 2006. Abbreviations: AR, Autosomal recessive; XL, X-linked; SCID, severe combined immunodeficiency; L, lymphocyte; M, monocyte; G, granulocyte; K, keratinocyte; NK, natural killer cell; P, polymorphonuclear cell (neutrophil); EC, endothelial cell; ICAM, intercellular adhesion molecule; TLR, toll-like receptors; ANC, absolute neutrophil count; G-CSF, granulocyte colony stimulating factor; rG-CSF, recombinant granulocyte colony stimulating factor; BMT, bone marrow transplant; Ig, immunoglobulin; DM, diabetes mellitus; FTT, failure to thrive; ↓, decreased; ↓↓, profoundly decreased; ↑, increased; N, normal; for Serum Ig Column: All refers to all isotypes.

Table VI. Complement pathway disorders

| Disorder | Abnormal gene | Abnormal genetic locus | Abnormal gene product | Classic/associated features | Functional defect | Inheritance |
|---|---|---|---|---|---|---|
| Classic complement pathway component defects | | | | | | |
| C1q α-chain deficiency<br>C1q β-chain deficiency<br>C1q γ-chain deficiency | *C1QA*<br>*C1QB*<br>*C1QG* | 1p36.3–p34.1 | C1q α chain<br>C1q β chain<br>C1q γ chain | SLE-like syndrome (often early-onset and may be severe), rheumatoid disease, pyogenic infections; defective classic complement pathway | Absent C-mediated hemolysis; impaired IC clearance | AR |
| C1r deficiency | *C1R* | 12p13 | C1r | SLE-like syndrome (often early-onset and may be severe), rheumatoid disease, pyogenic infections; defective classic complement pathway; often associated with C1s deficiency | Absent C-mediated hemolysis; impaired IC clearance | AR |
| C1s deficiency | *C1S* | 12p13 | C1s | SLE-like syndrome (often early-onset and may be severe), rheumatoid disease, pyogenic infections; defective classic complement pathway; often associated with C1r deficiency | Absent C-mediated hemolysis; impaired IC clearance | AR |
| C2 deficiency | *C2* | 6p21.3 | C2 | SLE and SLE-like syndrome (often less severe than in normocomplementemic patients), pyogenic encapsulated bacterial infections, vasculitis, Polymyositis, GN; most common C deficiency; C2 deficiency, Type 1 involves absence of C2, while Type 2 involves isolated secretion defect | Absent C-mediated hemolysis; impaired IC clearance | AR |
| C3 deficiency | *C3* | 19p13.3–p13.2 | C3 | Recurrent pyogenic infections, SLE, 25% with vasculitis or GN | Absent C-mediated hemolysis; impaired bactericidal action; absent opsonization; impaired IC clearance | AR |
| C4 deficiency | *C4A C4B* | 6p21.3 | C4a C4b | Pyogenic infections, SLE with or without GN; C4A and C4B both encode the C4 protein | Absent C-mediated hemolysis; impaired IC clearance | AR |
| Late complement pathway defects | | | | | | |
| C5 deficiency | *C5* | 9q32–q34 | C5 | Neisserial infections, SLE-like syndrome | Absent C-mediated hemolysis (defective MAC formation); impaired bactericidal action; | AR |
| C6 deficiency | *C6* | 5p13 | C6 | Neisserial infections, few with SLE-like syndrome | Absent C-mediated hemolysis (defective MAC formation); impaired bactericidal action; | AR |
| C7 deficiency | *C7* | 5p13 | C7 | Neisserial infections, SLE, rheumatoid arthritis, pyoderma gangrenosum, scleroderma, vasculitis | Absent C-mediated hemolysis (defective MAC formation); impaired bactericidal action; | AR |
| C8 α-chain deficiency | *C8A* | 1p32 | C8α | Neisserial infections, SLE-like syndrome, more common in African–Americans, abnormal C8α and normal C8γ chains covalently bind and then join normal C8α to form abnormal C8 | Absent C-mediated hemolysis (defective MAC formation); impaired bactericidal action; | AR |
| C8 β-chain deficiency | *C8B* | 1p32 | C8β | Neisserial infections, SLE-like syndrome, more common in caucasians | Absent C-mediated hemolysis (defective MAC formation); impaired bactericidal action; | AR |
| C8 γ-chain deficiency | *C8G* | 9q34.3 | C8γ | Neisserial infections, SLE-like syndrome, more common in African–Americans, normal C8α and abnormal C8γ chains covalently bind and then join normal C8α to form abnormal C8 | Absent C-mediated hemolysis (defective MAC formation); impaired bactericidal action; | AR |

Table VI – *continued*

| Disorder | Abnormal gene | Abnormal genetic locus | Abnormal gene product | Classic/associated features | Functional defect | Inheritance |
|---|---|---|---|---|---|---|
| C9 deficiency | *C9* | 5p13 | C9 | Often asymptomatic, some ability to kill Neisseria but slowly, some may still get systemic Neisserial infections, possess some serum hemolytic and bactericidal activity but less than normal, more common in Japan | Decreased C-mediated hemolysis (defective MAC formation); impaired bactericidal action | AR |
| Alternative complement pathway component defects | | | | | | |
| Factor B deficiency | *BF* | 6p21.3 | Factor B | Neisserial infections (meningococcemia) with high mortality, no detectable alternative pathway activity, decreased classical pathway activity and, only dysfunctional Factor B | Absent C-mediated hemolysis via alternate pathway | AR |
| Factor D deficiency | *DF* | 19p13.3 | Factor D | Neisserial infection, factor D converts factor B to Bb | Absent C-mediated hemolysis via alternate pathway | AR |
| Factor H deficiency | *HF1* | 1q32 | Factor H | Recurrent Neisserial and encapsulated bacterial infection; renal disease (GN, IgA nephropathy, hemolytic uremic syndrome); | Spontaneous activation of complement alternative pathway with C3 consumption via alternate pathway; factor H normally binds C3b and thus prevents Factor B from binding C3b and forming C3bBb (the alternative pathway C3 convertase); factor H also normally is a cofactor for Factor I, which inactivates C3b; factor H also increases dissociation of the C3 convertase (C3bBb) and C3bBb3b (C5 convertase) | AR |
| Properdin deficiency | *PFC* | Xp11.3–p11.23 | Properdin | Neisserial infections; properdin normally binds to and stabilizes the alternative pathway C3 and C5 convertases; may have complete deficiency (type I), incomplete deficiency (type II), or dysfunction of properdin (type III) | Absent C-mediated hemolysis via alternate pathway | XL |
| Mannose-binding lectin pathway component defects | | | | | | |
| Mannose-binding lectin deficiency | *MBL2* | 10q11.2–q21 | Mannose-binding lectin | Pyogenic infections; very low penetrance | Defective mannose recognition and lectin pathway-mediated hemolysis | AR |
| Mannose-binding lectin-associated serine protease (MASP) 2 deficiency | *MASP2* | 1p36.3–p36.2 | MASP2 | Pyogenic infections, SLE-like syndrome | Absent lectin pathway-mediated hemolysis | AR |
| Complement regulatory protein defects | | | | | | |
| C1 inhibitor deficiency (hereditary angioedema) | *SERPING1* | 11q12–q13.1 | C1 inhibitor | Hereditary Angioedema; episodic edema of the limbs, larynx, gastrointestinal tract; type 1 with no C1 Inhibitor, type 2 with normal or elevated levels of dysfunctional inhibitor | Spontaneous activation of classical pathway with consumption of C4/C2, blood coagulation, fibrinolysis, and kinin pathways; C1 inhibitor normally inhibits C1s, C1r, kallikrein conversion of high-molecular weight kininogen to bradykinin, plasmin, and Hageman factor autoactivation | AD |

Table VI – *continued*

| Disorder | Abnormal gene | Abnormal genetic locus | Abnormal gene product | Classic/associated features | Functional defect | Inheritance |
|---|---|---|---|---|---|---|
| C4 binding protein α deficiency | *C4BPA* | 1q32 | C4 bp α | Angioedema, cutaneous vasculitis, arthritis | Activation of classical pathway with consumption of C3, increased C3a and C5a anaphylatoxin formation; normally, C4 bp binds to and (by acting as a cofactor Factor I) degrades C4b; also, C4 bp accelerates the decay of C4b2a (classical pathway C3 convertase) | AR |
| C4 binding protein β deficiency | *C4BPB* | 1q32 | C4 bp β | Increased risk for thromboembolic disorders | Inactivation of the protein C anticoagulatory pathway; normally, C4 bp binds the anticoagulant vitamin K-dependent protein S; C4 bp β also is a part of the dimmer C4 bp | AR |
| Factor I deficiency | *IF* | 4q25 | Factor I | Recurrent pyogenic infections; some may be asymptomatic; rheumatic disease uncommon | Continuous activation and cleavage of C3 through the alternative pathway, producing C3b; normally Factor I cleaves C3b and C4b in presence of cofactors Factor H and C4 bp | AR |
| Decay-accelerating factor (CD55) deficiency | *DAF* | 1q32 | Decay accelerating factor | Paroxysmal Nocturnal Hemoglobinuria (increased lysis of red blood cells by complement); intravascular hemolysis, pancytopenia, and recurrent (usually venous) thromboses/Budd-Chiari syndrome; often in patients with aplastic anemia; possibility of transformation into acute myeloblastic leukemia. progressive renal impairment due to hemoglobinuria) | Abnormal activation of red cell lysis via the classical and alternative pathways; Normally, DAF, a control protein for the classical and alternative pathways, protects cells from complement-mediated cytolysis and is involved in T cell activation; DAF binds C3b and C4b, preventing their ability to activate C2 and Factor B. | AR |
| CD59 deficiency | *CD59* | 11p13 | CD59 | Recurrent hemolytic anemia and strokes; | MAC-mediated red blood cell lysis; Normally, CD59 is a potent inhibitor of MAC and involved in signal transduction for T cell activation and NK cell function | |

Data abstracted from Bonilla and Geha 2006, Buckley 2003a, Chapel et al. 2003, Anonymous 2000, Notarangelo et al. 2004, Vihinen 2004, Walport 2001a, Walport 2001b. Abbreviations: AR, Autosomal recessive; XL, X-linked; SCID, severe combined immunodeficiency; C, complement; IC, immune complex; SLE, systemic lupus erythematosus; GN, glomerulonephritis; MAC, membrane attack complex.

Table VII. Other well-defined immunodeficiency syndromes and PIDs.

| Disorder | Abnormal gene | Abnormal genetic locus | Abnormal gene product | Classic/associated features | T cell # (blood) | B cells # (blood) | Serum Ig | NK cell # (blood) | Inheritance |
|---|---|---|---|---|---|---|---|---|---|
| Wiskott–Aldrich syndrome | *WAS* | Xp11.4–p11.21 | WAS protein (WASP) | Thrombocytopenia; small, defective platelets; eczema; vasculitis; lymphoma; poor Ab to protein | ↓ progressive | N | Varies ↓ IgM, ↑ IgA, ↑ IgE | – | XL |
| DiGeorge syndrome | *DGCR* | 22q11 | multiple | Hypoparathyroidism, abnormal facies, conotruncal malformation; contiguous gene defect in 90% that affects thymic development | ↓ or N | N | N or ↓ | – | AD or *de novo* defect |
| Hyper IgE syndrome (Job's syndrome) | ? | ? | ? | Recurrent abscess formation in lungs, skin, joints, and other organs due primarily to Staphylococcus aureus, pneumatocele formation, and non-atopic dermatitis; coarse facies;dental and skeletal abnormalities; eosinophilia; decreased humoral (especially anamnestic) and cellular immune response to new antigens; AR type lack dental, skeletal, pneumatocele problems and have more severe viral infections | N | N | IgE ↑ ↑, IgD ↑ others N | – | AD or AR |
| X-linked lymphoproliferative syndrome (XLP) | *SH2D1A* | Xq25–q26 | SAP | Induced by EBV: Hepatitis, aplastic anemia, lymphoma | N | N or ↓ | N, rarely ↓ | – | XL |
| Autoimmune polyendocrinopathy–candidiasis–ectodermal dystrophy (APECED) | *AIRE* | 21q22.3 | AIRE | Autoimmune polyendocrinopathy, mucocutaneous candidiasis, ectodermal dysplasia | N | N | N | – | ? |
| Autoimmune Lymphoproliferative syndromes (ALPS) | | | | | | | | | |
| CD95 (Fas) Defect, ALPS type 1a | *TNFRSF6* | 10q23–q24.1 | Fas | Defective lymphocyte apoptosis; Splenomegaly, lymphadenopathy; risk of lymphoma; autoimmune cytopenias | N, ↑ double negative T cells, activated | N | N or ↑ | – | AR |
| CD95L (Fas ligand) Defect, ALPS type 1b | *TNFSF6* | 1q23–q23 | Fas Ligand | Defective apoptosis; lymphadenopathy; autoimmunity; lupus | N, ↑ double negative T cells | N | N | – | AR |
| Caspase 10 Defect, ALPS type 2a | *CASP10* | 2q33–q34 | Caspase 10 | Defective apoptosis; Splenomegaly, lymphadenopathy; autoimmunityl increased dendritic cells | N, ↑ double negative T cells | N | N | – | AR |
| Caspase 8 Defect, ALPS type 2b | *CASP8* | 2q33–q34 | Caspase 8 | Defective lymphocyte apoptosis and activation; Splenomegaly, lymphadenopathy; recurrent bacterial and viral infection | N, slightly ↑ double negative T cells; ↓ $CD4^{+}T$ | N | N or ↓ | – | AR |

Table VII – *continued*

| Disorder | Abnormal gene | Abnormal genetic locus | Abnormal gene product | Classic/associated features | T cell # (blood) | B cells # (blood) | Serum Ig | NK cell # (blood) | Inheritance |
|---|---|---|---|---|---|---|---|---|---|
| Familial Hemophagocytic Lymphohistiocytosis (FHPL) | | | | | | | | | |
| FHPL type 1 (FHPL1) | ? | 9q21.3–q22 | ? | Early onset, multisystem, fatal immunoregulatory disorder with uncontrolled activation of T cells and macrophages (hemophagocytic activation) that infiltrate liver, spleen, bone marrow, CNS; highly variable symptoms include fever, irritability, general pain, edema, hepatosplenomegaly, rash, neurological problems; granulocytopenia, thrombocytopenia, anemia are due to phagocytosis of these cells and histiocytic infiltration of the bone marrow; often have a healthy few months followed by a viral infection-triggered (usually) hemophagocytic activation phase; immunosuppressants, steroids, and other agents may precede BMT | N | N | N | – | ? |
| Perforin deficiency (FHPL type 2, FHPL2) | *PRF1* | 10q22 | Perforin | Same as FHPL1 ↓NK and CTL activity; due to defects in perforin, which polymerizes into transmembrane channels that lyse cells | N | N | N | – | AR |
| Munc deficiency (FHPL type 3, FHPL3) | *UNC13D* | 17q25.3 | UNC13d | Same as FHPL1; ↓NK and CTL activity; due to defects in UNC13d, which is involved in vesicle maturation during exocytosis of cytolytic granule contents | N | N | N | – | AR |
| STX11 deficiency (FHPL type 4, FHPL4) | *STX11* | 6q24 | STX11 | Same as FHPL1; due to defect in STX11, which like UNC13dl, functions in membrane trafficking and vesicle fusion | N | N | N | – | AR |
| Others | | | | | | | | | |
| Immunodeficiency, Polyendocrinopathy, Enteropathy, X-linked Syndrome (IPEX) | *FOXP3* | Xp11.23–q13.3 | FOXP3 | Infections; prolonged diarrhea; ichthyosiform dermatitis, early-onset IDDM, thyroiditis; hemolytic anemia, variable autoimmune phenomena; often fatal in infancy or early childhood; supportive treatment and BMT are treatment options; FOXP3 is a homologue of the murine protein, Scurfin, which is required for the development of $CD4^+$ $CD25^+$ T regulatory cells. Without these T cells, CD4 cells trigger tissue damage | – | – | – | – | XL |

Table VII – *continued*

| Disorder | Abnormal gene | Abnormal genetic locus | Abnormal gene product | Classic/associated features | T cell # (blood) | B cells # (blood) | Serum Ig | NK cell # (blood) | Inheritance |
|---|---|---|---|---|---|---|---|---|---|
| Cartilage–Hair Hypoplasia | *RMRP* | 9p21–p12 | RMRP (RNA component of mitochondrial RNA-processing endoribo-nuclease) | Progressive metaphyseal chrondrodysplasia causing short stature, short hands, possibly short deformed limbs; fine, slow-growing hair; Neutropenia; immunodeficiency in many, especially with varicella | ↓ | ↓ | ↓ or N | – | AR |

Data abstracted from Bennett et al. 2001, Bennett and Ochs 2001, Bonilla and Geha 2006, Buckley 2003a, Chapel et al. 2003, Anonymous 2000, Notarangelo et al. 2004, Online Mendelian Inheritance in Man 2000, Sole et al. 1993, Vihinen 2004, Wildin et al. 2002, zur Stadt et al. 2005. Abbreviations: AR, Autosomal recessive; XL, X-linked; SCID, severe combined immunodeficiency; CNS, central nervous system; ↓, decreased; ↓↓, profoundly decreased; ↑, increased; N, normal; for serum Ig Column: All refers to All isotypes.

defective in XLP (Sayos et al. 1998, Morra et al. 2001). Without SAP, EBV infection-induced T cell proliferation is uncontrolled in XLP, and NK cell function is abnormal. HLA-identical or unrelated BMT has cured the disease in about half of known attempts (Gross et al. 1996; Table VII).

*Autoimmune polyendocrinopathy–candidiasis–ectodermal dystrophy (APECED)*

APECED is a subset of the group of disorders known as CMCC. APECED is also known as autoimmune polyglandular syndrome 1 (APS-1). APECED is associated with a mutation in the autoimmune regulator (AIRE), a molecule of uncertain function. The disease is characterized by autoimmunity against numerous endocrine organs resulting in hypothyroidism, hypoparathyroidism, adrenocortical failure, insulin-dependent diabetes mellitus, hypogonadism, pernicious anemia, hepatitis, CMCC, and ectodermal problems (vitiligo, alopecia, dental enamel dystrophy) (Aaltonen and Bjorses 1999, Vihinen 2004, Villasenor et al. 2005). Candidiasis typically requires antifungal therapy (Table VII). Descriptions of additional PID syndromes can be found in Table VII.

### Diagnostic considerations (Buckley 1986, Tangsinmankong et al. 2001, Bonilla et al. 2005)

The medical history should be thorough but special attention should be given to types, frequency, and severity of infections; age of onset, presence of atopic disease, systemic transfusion reactions, and risk factors or evidence for secondary immunodeficiency. Family history should address infections or early deaths in other family members, as well as parental consanguinity. Physical exam should look for signs specific to the various immunodeficiencies including presence or absence of tonsillar or peripheral lymphoid tissue (e.g. XLA), skin or nail changes (e.g. AT, EDA-ID, Chediak–Higashi syndrome), severe eczema (e.g. WAS, hyper IgE syndrome), abnormal facies (e.g. DGS, Bloom syndrome, ICF, NBS, hyper IgE syndrome), short stature (e.g. DNA repair defects), cardiac anomalies (DGS), and neurologic deficits (e.g. AT, PNP deficiency). Radiology studies may detect an absent thymic shadow in infants with DGS or SCID, skeletal abnormalities in patients with ADA deficiency, pneumatoceles in hyper IgE syndrome, great vessel anomalies in DGS, and other such disease specific findings. Minimal basic lab evaluation should include complete blood count with manual differential, and, as appropriate, evaluation of humoral and cellular immunity.

Humoral immune evaluation typically includes serum Ig levels with isotypes; measurement of specific antibody response to polysaccharides (isohemagglutinins to ABO blood groups, Pneumovax) and proteins

(conjugated pneumococcal vaccine, conjugated *Hemophilus influenza B* vaccine, diphtheria toxoid, tetanus toxoid); and quantitation of B cell numbers and markers with flow cytometry.

If infection pattern or other evidence suggests cellular PID, T cell numbers and subsets can be measured with flow cytometry or monoclonal antibody methods. Alternatively, delayed hypersensitivity skin tests against common antigens such as Candida, tetanus toxoid, mumps, Trychophyton and tuberculin, can be performed for assessment of cellular immune function. Non-specific and specific *in vitro* stimulation of T cell proliferation may provide more specific information in some cases. Should phagocyte dysfunction be suspected, in addition to measuring the absolute numbers of and assessing the morphology of circulating neutrophils with the manual differential, the NBT reduction test or chemiluminescence/flow cytometry respiratory burst assays can assess neutrophil oxidative capacity.

Overall complement function can be evaluated using the complement hemolytic assays (CH50 for the classic pathway and AH50 for the alternative pathway), while individual complement components in the blood can be quantified in local or specialty labs. Selection of tests should be individualized and interpreted with the clinical context in mind. Abnormal tests should be repeated. Genetic testing for individual PIDs may be pursued at specialty labs or immunodeficiency centers and is encouraged to increase the base of knowledge of these rare disorders.

In X-linked disease, heterozygous female carriers may be detected by looking for non-random patterns of X-chromosome inactivation in the affected cells type (Bonilla and Geha 2003, Bonilla et al. 2005). Prenatal diagnosis is also possible in high-risk pregnancies, using umbilical cord blood lymphocytes for phenotype and functional analysis. If the gene defect is known, genetic tests are appropriate.

### Conclusion

Clearly, PIDs are uncommon disorders. Despite this, the last few decades have seen an explosion in study of primary immunodeficiency and better understanding of the basic defects in many of them. This rapid pace of study and growing knowledge of the mechanisms of immunity in health and disease will continue and hopefully will result in better treatments and healthier lives for patients with immunodeficiency.

### References

Aaltonen J, Bjorses P. 1999. Cloning of the APECED gene provides new insight into human autoimmunity. Ann Med 31:111–116.

Aghamohammadi A, Moin M, Farhoudi A, Rezaei N, Pourpak Z, Movahedi M, Gharagozlou M, Nabavi M, Shahrokhi A. 2004. Efficacy of intravenous immunoglobulin on the prevention of pneumonia in patients with agammaglobulinemia. FEMS Immunol Med Microbiol 40:113–118.

Aiuti A. 2004. Gene therapy for adenosine–deaminase-deficient severe combined immunodeficiency. Best Pract Res Clin Haematol 17:505–516.

Aiuti A, Slavin S, Aker M, Ficara F, Deola S, Mortellaro A, Morecki S, Andolfi G, Tabucchi A, Carlucci F, Marinello E, Cattaneo F, Vai S, Servida P, Miniero R, Roncarolo MG, Bordignon C. 2002. Correction of ADA–SCID by stem cell gene therapy combined with nonmyeloablative conditioning. Science 296:2410–2413.

Aleman K, Noordzij JG, de Groot R, van Dongen JJ, Hartwig NG. 2001. Reviewing Omenn syndrome. Eur J Pediatr 160:718–725.

Alyanakian MA, Bernatowska E, Scherrmann JM, Aucouturier P, Poplavsky JL. 2003. Pharmacokinetics of total immunoglobulin G and immunoglobulin G subclasses in patients undergoing replacement therapy for primary immunodeficiency syndromes. Vox Sang 84:188–192.

Anonymous, 2000. Nlm: Online Mendelian Inheritance in Man, OMIM. Baltimore, MD and Bethesda, MD, McKusick–Nathans Institute for Genetic Medicine, Johns Hopkins University and National Center for Biotechnology Information, National Library of Medicine.

Antall PM, Meyerson H, Kaplan D, Venglarcik J, Hostoffer RW. 1999. Selective antipolysaccharide antibody deficiency associated with peripheral blood CD5 + B-cell predominance. J Allergy Clin Immunol 103:637–641.

Asao H, Okuyama C, Kumaki S, Ishii N, Tsuchiya S, Foster D, Sugamura K. 2001. Cutting edge: The common gamma-chain is an indispensable subunit of the IL-21 receptor complex. J Immunol 167:1–5.

Ashman RF, Schaffer FM, Kemp JD, Yokoyama WM, Zhu ZB, Cooper MD, Volanakis JE. 1992. Genetic and immunologic analysis of a family containing five patients with common-variable immune deficiency or selective IgA deficiency. J Clin Immunol 12:406–414.

Auricchio L, Adriani M, Frank J, Busiello R, Christiano A, Pignata C. 2005. Nail dystrophy associated with a heterozygous mutation of the nude/SCID human FOXN1 (WHN) gene. Arch Dermatol 141:647–648.

Bennett CL, Christie J, Ramsdell F, Brunkow ME, Ferguson PJ, Whitesell L, Kelly TE, Saulsbury FT, Chance PF, Ochs HD. 2001. The immune dysregulation, polyendocrinopathy, enteropathy, X-linked syndrome (IPEX) is caused by mutations of FOXP3. Nat Genet 27:20–21.

Bennett CL, Ochs HD. 2001. IPEX is a unique X-linked syndrome characterized by immune dysfunction, polyendocrinopathy, enteropathy, and a variety of autoimmune phenomena. Curr Opin Pediatr 13:533–538.

Blanco-Betancourt CE, Moncla A, Milili M, Jiang YL, Viegas-Pequignot EM, Roquelaure B, Thuret I, Schiff C. 2004. Defective B-cell-negative selection and terminal differentiation in the ICF syndrome. Blood 103:2683–2690.

Bonilla FA, Bernstein IL, Khan DA, Ballas ZK, Chinen J, Frank MM, Kobrynski LJ, Levinson AI, Mazer B, Nelson RP, Jr, Orange JS, Routes JM, Shearer WT, Sorensen RU. 2005. Practice parameter for the diagnosis and management of primary immunodeficiency. Ann Allergy Asthma Immunol 94:S1–63.

Bonilla FA, Geha RS. 2003. 12. Primary immunodeficiency diseases. J Allergy Clin Immunol 111:S571–S581.

Bonilla FA, Geha RS. 2006. 2. Update on primary immunodeficiency diseases. J Allergy Clin Immunol 117:S435–S441.

Buckley RH. 1986. Advances in the diagnosis and treatment of primary immunodeficiency diseases. Arch Intern Med 146:377–384.

Buckley RH. 2003a. Primary immunodeficiency diseases. In: AdkinsonNF, Jr, Bochner BS, Yunginger JW, Holgate ST, Busse WW, Simons FER, editors. Middleton's allergy principles and practice. 6. Philadelphia, PA: Mosby. p 1015–1042.

Buckley RH. 2003b. Treatment options for genetically determined immunodeficiency. Lancet 361:541–542.

Buckley RH, Schiff SE, Schiff RI, Markert L, Williams LW, Roberts JL, Myers LA, Ward FE. 1999. Hematopoietic stem-cell transplantation for the treatment of severe combined immunodeficiency. N Engl J Med 340:508–516.

Buckley RH, Schiff SE, Schiff RI, Roberts JL, Markert ML, Peters W, Williams LW, Ward FE. 1993. Haploidentical bone marrow stem cell transplantation in human severe combined immunodeficiency. Semin Hematol 30(92–101):2–4.

Burks AW, Sampson HA, Buckley RH. 1986. Anaphylactic reactions after gamma globulin administration in patients with hypogammaglobulinemia. Detection of IgE antibodies to IgA. N Engl J Med 314:560–564.

Buzi F, Notarangelo LD, Plebani A, Duse M, Parolini O, Monteleone M, Ugazio AG. 1994. X-linked agammaglobulinemia, growth hormone deficiency and delay of growth and puberty. Acta Paediatr 83:99–102.

Castigli E, Pahwa R, Good RA, Geha RS, Chatila TA. 1993. Molecular basis of a multiple lymphokine deficiency in a patient with severe combined immunodeficiency. Proc Natl Acad Sci U S A 90:4728–4732.

Cham B, Bonilla MA, Winkelstein J. 2002. Neutropenia associated with primary immunodeficiency syndromes. Semin Hematol 39:107–112.

Chan AC, Kadlecek TA, Elder ME, Filipovich AH, Kuo WL, Iwashima M, Parslow TG, Weiss A. 1994. ZAP-70 deficiency in an autosomal recessive form of severe combined immunodeficiency. Science 264:1599–1601.

Chapel H, Geha R, Rosen F. 2003. Primary immunodeficiency diseases: An update. Clin Exp Immunol 132:9–15.

Chinen J, Puck JM. 2004. Perspectives of gene therapy for primary immunodeficiencies. Curr Opin Allergy Clin Immunol 4:523–527.

Clark JA, Callicoat PA, Brenner NA, Bradley CA, Smith DM, Jr. 1983. Selective IgA deficiency in blood donors. Am J Clin Pathol 80:210–213.

Cleveland WW. 1975. Immunologic reconstitution in the DiGeorge syndrome by fetal thymic transplant. Birth Defects Orig Artic Ser 11:352–356.

Conley ME, Notarangelo LD, Etzioni A. 1999. Diagnostic criteria for primary immunodeficiencies. Representing PAGID (Pan–American Group for Immunodeficiency) and ESID (European Society for Immunodeficiencies). Clin Immunol 93:190–197.

Conley ME, Saragoussi D, Notarangelo L, Etzioni A, Casanova JL. 2003. An international study examining therapeutic options used in treatment of Wiskott–Aldrich syndrome. Clin Immunol 109:272–277.

Corneo B, Moshous D, Gungor T, Wulffraat N, Philippet P, Le Deist FL, Fischer A, de Villartay JP. 2001. Identical mutations in RAG1 or RAG2 genes leading to defective V(D)J recombinase activity can cause either T–B-severe combined immune deficiency or Omenn syndrome. Blood 97:2772–2776.

Courtois G, Smahi A, Reichenbach J, Doffinger R, Cancrini C, Bonnet M, Puel A, Chable-Bessia C, Yamaoka S, Feinberg J, Dupuis-Girod S, Bodemer C, Livadiotti S, Novelli F, Rossi P, Fischer A, Israel A, Munnich A, Le Deist F, Casanova JL. 2003. A hypermorphic IkappaBalpha mutation is associated with autosomal dominant anhidrotic ectodermal dysplasia and T cell immunodeficiency. J Clin Invest 112:1108–1115.

Cunningham-Rundles C. 1989. Clinical and immunologic analyses of 103 patients with common variable immunodeficiency. J Clin Immunol 9:22–33.

Cunningham-Rundles C. 2001. Physiology of IgA and IgA deficiency. J Clin Immunol 21:303–309.

Czarnetzki BM. 1989. Disorders of phagocyte killing and digestion (CGD, G-6-PD and myeloperoxidase deficiencies). Curr Probl Dermatol 18:101–105.

de la Calle-Martin O, Hernandez M, Ordi J, Casamitjana N, Arostegui JI, Caragol I, Ferrando M, Labrador M, Rodriguez-Sanchez JL, Espanol T. 2001. Familial CD8 deficiency due to a mutation in the CD8 alpha gene. J Clin Invest 108:117–123.

de la Salle H, Hanau D, Fricker D, Urlacher A, Kelly A, Salamero J, Powis SH, Donato L, Bausinger H, Laforet M, et al. 1994. Homozygous human TAP peptide transporter mutation in HLA class I deficiency. Science 265:237–241.

de Vries E, Koene HR, Vossen JM, Gratama JW, von dem Borne AE, Waaijer JL, Haraldsson A, de Haas M, van Tol MJ. 1996. Identification of an unusual Fc gamma receptor IIIa (CD16) on natural killer cells in a patient with recurrent infections. Blood 88:3022–3027.

de Weers M, Verschuren MC, Kraakman ME, Mensink RG, Schuurman RK, van Dongen JJ, Hendriks RW. 1993. The Bruton's tyrosine kinase gene is expressed throughout B cell differentiation, from early precursor B cell stages preceding immunoglobulin gene rearrangement up to mature B cell stages. Eur J Immunol 23:3109–3114.

Devriendt K, Kim AS, Mathijs G, Frints SG, Schwartz M, Van Den Oord JJ, Verhoef GE, Boogaerts MA, Fryns JP, You D, Rosen MK, Vandenberghe P. 2001. Constitutively activating mutation in WASP causes X-linked severe congenital neutropenia. Nat Genet 27:313–317.

DiSanto JP, Bonnefoy JY, Gauchat JF, Fischer A, de Saint Basile G. 1993. CD40 ligand mutations in x-linked immunodeficiency with hyper-IgM. Nature 361:541–543.

DiSanto JP, Keever CA, Small TN, Nicols GL, O'Reilly RJ, Flomenberg N. 1990. Absence of interleukin 2 production in a severe combined immunodeficiency disease syndrome with T cells. J Exp Med 171:1697–1704.

Doffinger R, Jouanguy E, Altare F, Wood P, Shirakawa T, Novelli F, Lammas D, Kumararatne D, Casanova JL. 1999. Inheritable defects in interleukin-12- and interferon-gamma-mediated immunity and the TH1/TH2 paradigm in man. Allergy 54:409–412.

Dogu F, Ikinciogullari A, Babacan E. 2004. Transient hypogammaglobulinemia of infancy and early childhood: Outcome of 30 cases. Turk J Pediatr 46:120–124.

Durandy A, Revy P, Fischer A. 2003. Hyper-immunoglobulin-M syndromes caused by an intrinsic B cell defect. Curr Opin Allergy Clin Immunol 3:421–425.

Eisenstein EM, Sneller MC. 1994. Common variable immunodeficiency: Diagnosis and management. Ann Allergy 73:285–292, quiz 93–4.

Elder ME, Lin D, Clever J, Chan AC, Hope TJ, Weiss A, Parslow TG. 1994. Human severe combined immunodeficiency due to a defect in ZAP-70, a T cell tyrosine kinase. Science 264:1596–1599.

Farrant J, Spickett G, Matamoros N, Copas D, Hernandez M, North M, Chapel H, Webster AD. 1994. Study of B and T cell phenotypes in blood from patients with common variable immunodeficiency (CVID). Immunodeficiency 5:159–169.

Farrington M, Grosmaire LS, Nonoyama S, Fischer SH, Hollenbaugh D, Ledbetter JA, Noelle RJ, Aruffo A, Ochs HD. 1994. CD40 ligand expression is defective in a subset of patients with common variable immunodeficiency. Proc Natl Acad Sci U S A 91:1099–1103.

Ferguson C, Larochelle A, Dunbar CE. 2005. Hematopoietic stem cell gene therapy: Dead or alive? Trends Biotechnol 23:589–597.

Feske S, Muller JM, Graf D, Kroczek RA, Drager R, Niemeyer C, Baeuerle PA, Peter HH, Schlesier M. 1996. Severe combined immunodeficiency due to defective binding of the nuclear factor of activated T cells in T lymphocytes of two male siblings. Eur J Immunol 26:2119–2126.

Filipovich AH, Mathur A, Kamat D, Kersey JH, Shapiro RS. 1994. Lymphoproliferative disorders and other tumors complicating immunodeficiencies. Immunodeficiency 5:91–112.

Fischer A, Hacein-Bey-Abina S, Cavazzana-Calvo M. 2004. Gene therapy for immunodeficiency diseases. Semin Hematol 41:272–278.

Gadola SD, Moins-Teisserenc HT, Trowsdale J, Gross WL, Cerundolo V. 2000. TAP deficiency syndrome. Clin Exp Immunol 121:173–178.

Gaspar HB, Parsley KL, Howe S, King D, Gilmour KC, Sinclair J, Brouns G, Schmidt M, Von Kalle C, Barington T, Jakobsen MA, Christensen HO, Al Ghonaium A, White HN, Smith JL, Levinsky RJ, Ali RR, Kinnon C, Thrasher AJ. 2004. Gene therapy of X-linked severe combined immunodeficiency by use of a pseudotyped gammaretroviral vector. Lancet 364:2181–2187.

Goldacker S, Warnatz K. 2005. Tackling the heterogeneity of CVID. Curr Opin Allergy Clin Immunol 5:504–509.

Grimbacher B, Holland SM, Gallin JI, Greenberg F, Hill SC, Malech HL, Miller JA, O'Connell AC, Puck JM. 1999. Hyper-IgE syndrome with recurrent infections–an autosomal dominant multisystem disorder. N Engl J Med 340:692–702.

Grimbacher B, Holland SM, Puck JM. 2005. Hyper-IgE syndromes. Immunol Rev 203:244–250.

Grimbacher B, Warnatz K, Peter HH. 2003. The immunological synapse for B-cell memory: the role of the ICOS and its ligand for the longevity of humoral immunity. Curr Opin Allergy Clin Immunol 3:409–419.

Gross TG, Filipovich AH, Conley ME, Pracher E, Schmiegelow K, Verdirame JD, Vowels M, Williams LL, Seemayer TA. 1996. Cure of X-linked lymphoproliferative disease (XLP) with allogeneic hematopoietic stem cell transplantation (HSCT): Report from the XLP registry. Bone Marrow Transplant 17:741–744.

Hammarstrom L, Vorechovsky I, Webster D. 2000. Selective IgA deficiency (SIgAD) and common variable immunodeficiency (CVID). Clin Exp Immunol 120:225–231.

Hermaszewski R, Ratnavel R, Webster AD, Denman AM. 1993. Rheumatoid arthritis in a patient with primary hypogammaglobulinaemia. Br J Rheumatol 32:636–639.

Javier FC, 3rd, Moore CM, Sorensen RU. 2000. Distribution of primary immunodeficiency diseases diagnosed in a pediatric tertiary hospital. Ann Allergy Asthma Immunol 84:25–30.

Kaneko H, Katagiri-Kawade M, Motoyoshi F, Tashita H, Teramoto T, Kondo N. 1996. Abnormal B cell response of protein kinase C in some common variable immunodeficiency. Exp Clin Immunogenet 13:36–42.

Kaneko H, Kondo N. 2004. Clinical features of Bloom syndrome and function of the causative gene, BLM helicase. Expert Rev Mol Diagn 4:393–401.

Kilic SS, Tezcan I, Sanal O, Metin A, Ersoy F. 2000. Transient hypogammaglobulinemia of infancy: Clinical and immunologic features of 40 new cases. Pediatr Int 42:647–650.

Kim MS, Basson CT. 2001. Wrapping up DiGeorge syndrome in a T-box? Pediatr Res 50:307–308.

Kung C, Pingel JT, Heikinheimo M, Klemola T, Varkila K, Yoo LI, Vuopala K, Poyhonen M, Uhari M, Rogers M, Speck SH, Chatila T, Thomas ML. 2000. Mutations in the tyrosine phosphatase CD45 gene in a child with severe combined immunodeficiency disease. Nat Med 6:343–345.

Lavilla P, Gil A, Rodriguez MC, Dupla ML, Pintado V, Fontan G. 1993. X-linked agammaglobulinemia and gastric adenocarcinoma. Cancer 72:1528–1531.

Lee AH, Levinson AI, Schumacher Jr, HR. 1993. Hypogammaglobulinemia and rheumatic disease. Semin Arthritis Rheum 22:252–264.

Levitt D, Haber P, Rich K, Cooper MD. 1983. Hyper IgM immunodeficiency. A primary dysfunction of B lymphocyte isotype switching. J Clin Invest 72:1650–1657.

Li L, Moshous D, Zhou Y, Wang J, Xie G, Salido E, Hu D, de Villartay JP, Cowan MJ. 2002. A founder mutation in Artemis, an SNM1-like protein, causes SCID in Athabascan-speaking Native Americans. J Immunol 168:6323–6329.

Lilic D. 2002. New perspectives on the immunology of chronic mucocutaneous candidiasis. Curr Opin Infect Dis 15:143–147.

Lindegren ML, Kobrynski L, Rasmussen SA, Moore CA, Grosse SD, Vanderford ML, Spira TJ, McDougal JS, Vogt RF, Jr, Hannon WH, Kalman LV, Chen B, Mattson M, Baker TG, Khoury M. 2004. Applying public health strategies to primary immunodeficiency diseases: A potential approach to genetic disorders. MMWR Recomm Rep 53:1–29.

Litwin SD. 1979. Immunodeficiency with thymoma: Failure to induce Ig production in immunodeficient lymphocytes cocultured with normal T cells. J Immunol 122:728–732.

Mamishi S, Zomorodian K, Saadat F, Gerami-Shoar M, Tarazooie B, Siadati SA. 2005. A case of invasive aspergillosis in CGD patient successfully treated with Amphotericin B and INF-gamma. Ann Clin Microbiol Antimicrob 4:4.

Marone G, Albini F, di Martino L, Quattrin S, Poto S, Condorelli M. 1986. The Wiskott–Aldrich syndrome: Studies of platelets, basophils and polymorphonuclear leucocytes. Br J Haematol 62:737–745.

Matamoros Flori N, Mila Llambi J, Espanol Boren T, Raga Borja S, Fontan Casariego G. 1997. Primary immunodeficiency syndrome in Spain: First report of the National registry in children and adults. J Clin Immunol 17:333–339.

Mellor DH. 1981. Virus infections of the central nervous system in children with primary immune deficiency disorders. Dev Med Child Neurol 23:807–810.

Minegishi Y, Coustan-Smith E, Wang YH, Cooper MD, Campana D, Conley ME. 1998. Mutations in the human lambda5/14.1 gene result in B cell deficiency and agammaglobulinemia. J Exp Med 187:71–77.

Minegishi Y, Rohrer J, Coustan-Smith E, Lederman HM, Pappu R, Campana D, Chan AC, Conley ME. 1999. An essential role for BLNK in human B cell development. Science 286:1954–1957.

Morell A. 1994. Clinical relevance of IgG subclass deficiencies. Ann Biol Clin (Paris) 52:49–52.

Morimoto Y, Routes JM. 2005. Granulomatous disease in common variable immunodeficiency. Curr Allergy Asthma Rep 5:370–375.

Morra M, Howie D, Grande MS, Sayos J, Wang N, Wu C, Engel P, Terhorst C. 2001. X-linked lymphoproliferative disease: A progressive immunodeficiency. Annu Rev Immunol 19:657–682.

Myers LA, Hershfield MS, Neale WT, Escolar M, Kurtzberg J. 2004. Purine nucleoside phosphorylase deficiency (PNP-def) presenting with lymphopenia and developmental delay: Successful correction with umbilical cord blood transplantation. J Pediatr 145:710–712.

Niehues T, Reichenbach J, Neubert J, Gudowius S, Puel A, Horneff G, Lainka E, Dirksen U, Schroten H, Doffinger R, Casanova JL, Wahn V. 2004. Nuclear factor kappaB essential modulator-deficient child with immunodeficiency yet without anhidrotic ectodermal dysplasia. J Allergy Clin Immunol 114:1456–1462.

Nonoyama S, Farrington M, Ishida H, Howard M, Ochs HD. 1993. Activated B cells from patients with common variable immunodeficiency proliferate and synthesize immunoglobulin. J Clin Invest 92:1282–1287.

Notarangelo L, Casanova JL, Fischer A, Puck J, Rosen F, Seger R, Geha R. 2004. Primary immunodeficiency diseases: An update. J Allergy Clin Immunol 114:677–687.

Notarangelo LD, Mazza C, Giliani S, D'Aria C, Gandellini F, Ravelli C, Locatelli MG, Nelson DL, Ochs HD, Notarangelo LD. 2002. Missense mutations of the WASP gene cause intermittent X-linked thrombocytopenia. Blood 99:2268–2269.

O'Connell AC, Puck JM, Grimbacher B, Facchetti F, Majorana A, Gallin JI, Malech HL, Holland SM. 2000. Delayed eruption of permanent teeth in hyperimmunoglobulinemia E recurrent infection syndrome. Oral Surg Oral Med Oral Pathol Oral Radiol Endod 89:177–185.

Oda A, Ochs HD. 2000. Wiskott-Aldrich syndrome protein and platelets. Immunol Rev 178:111–117.

Online Mendelian Inheritance in Man, OMIM In Baltimore, MD and Bethesda, MD, McKusick-Nathans Institute for Genetic Medicine, Johns Hopkins University and National Center for Biotechnology Information, National Library of Medicine, 2000.

Orange JS. 2002. Human natural killer cell deficiencies and susceptibility to infection. Microbes Infect 4:1545–1558.

Orange JS, Jain A, Ballas ZK, Schneider LC, Geha RS, Bonilla FA. 2004a. The presentation and natural history of immunodeficiency caused by nuclear factor kappaB essential modulator mutation. J Allergy Clin Immunol 113:725–733.

Orange JS, Levy O, Brodeur SR, Krzewski K, Roy RM, Niemela JE, Fleisher TA, Bonilla FA, Geha RS. 2004b. Human nuclear factor kappa B essential modulator mutation can result in immunodeficiency without ectodermal dysplasia. J Allergy Clin Immunol 114:650–656.

Paller AS. 1995. Immunodeficiency syndromes. X-linked agammaglobulinemia, common variable immunodeficiency, Chediak–Higashi syndrome, Wiskott–Aldrich syndrome, and X-linked lymphoproliferative disorder. Dermatol Clin 13:65–71.

Perlman S, Becker-Catania S, Gatti RA. 2003. Ataxia–telangiectasia: Diagnosis and treatment. Semin Pediatr Neurol 10:173–182.

Rane SG, Reddy EP. 2000. Janus kinases: Components of multiple signaling pathways. Oncogene 19:5662–5679.

Resta R, Thompson LF. 1997. SCID: The role of adenosine deaminase deficiency. Immunol Today 18:371–374.

Revy P, Muto T, Levy Y, Geissmann F, Plebani A, Sanal O, Catalan N, Forveille M, Dufourcq-Labelouse R, Gennery A, Tezcan I, Ersoy F, Kayserili H, Ugazio AG, Brousse N, Muramatsu M, Notarangelo LD, Kinoshita K, Honjo T, Fischer A, Durandy A. 2000. Activation-induced cytidine deaminase (AID) deficiency causes the autosomal recessive form of the Hyper-IgM syndrome (HIGM2). Cell 102:565–575.

Riminton DS, Limaye S. 2004. Primary immunodeficiency diseases in adulthood. Intern Med J 34:348–354.

Roper M, Parmley RT, Crist WM, Kelly DR, Cooper MD. 1985. Severe congenital leukopenia (reticular dysgenesis). Immunologic and morphologic characterizations of leukocytes. Am J Dis Child 139:832–835.

Rosefsky JB. 1990. Transient hypogammaglobulinemia of infancy. Acta Paediatr Scand 79:962–963.

Salzer U, Chapel HM, Webster AD, Pan-Hammarstrom Q, Schmitt-Graeff A, Schlesier M, Peter HH, Rockstroh JK, Schneider P, Schaffer AA, Hammarstrom L, Grimbacher B. 2005. Mutations in TNFRSF13B encoding TACI are associated with common variable immunodeficiency in humans. Nat Genet 37:820–828.

Salzer U, Grimbacher B. 2005. TACItly changing tunes: Farewell to a yin and yang of BAFF receptor and TACI in humoral immunity? New genetic defects in common variable immunodeficiency. Curr Opin Allergy Clin Immunol 5:496–503.

Salzer U, Maul-Pavicic A, Cunningham-Rundles C, Urschel S, Belohradsky BH, Litzman J, Holm A, Franco JL, Plebani A, Hammarstrom L, Skrabl A, Schwinger W, Grimbacher B. 2004. ICOS deficiency in patients with common variable immunodeficiency. Clin Immunol 113:234–240.

Sandler SG. 2006. How I manage patients suspected of having had an IgA anaphylactic transfusion reaction. Transfusion 46:10–13.

Sandler SG, Mallory D, Malamut D, Eckrich R. 1995. IgA anaphylactic transfusion reactions. Transfus Med Rev 9:1–8.

Sandler SG, Trimble J, Mallory DM. 1996. Coexistent IgG2 and IgA deficiencies in blood donors. Transfusion 36:256–258.

Sandler SG, Zantek ND. 2004. Review: IgA anaphylactic transfusion reactions. Part II. Clinical diagnosis and bedside management. Immunohematology 20:234–238.

Sany J, Jorgensen CH, Anaya JM, Didry C, Andary M, Serre I, Baldet P. 1993. Arthritis associated with primary agammaglobulinemia: New clues to its immunopathology. Clin Exp Rheumatol 11:65–69.

Sawada A, Takihara Y, Kim JY, Matsuda-Hashii Y, Tokimasa S, Fujisaki H, Kubota K, Endo H, Onodera T, Ohta H, Ozono K, Hara J. 2003. A congenital mutation of the novel gene LRRC8 causes agammaglobulinemia in humans. J Clin Invest 112:1707–1713.

Sayos J, Wu C, Morra M, Wang N, Zhang X, Allen D, van Schaik S, Notarangelo L, Geha R, Roncarolo MG, Oettgen H, De Vries JE, Aversa G, Terhorst C. 1998. The X-linked lymphoproliferative-disease gene product SAP regulates signals induced through the co-receptor SLAM. Nature 395:462–469.

Seyama K, Nonoyama S, Gangsaas I, Hollenbaugh D, Pabst HF, Aruffo A, Ochs HD. 1998. Mutations of the CD40 ligand gene and its effect on CD40 ligand expression in patients with X-linked hyper IgM syndrome. Blood 92:2421–2434.

Shackelford PG. 1993. IgG subclasses: Importance in pediatric practice. Pediatr Rev 14:291–296.

Shackelford PG, Granoff DM, Madassery JV, Scott MG, Nahm MH. 1990a. Clinical and immunologic characteristics of healthy children with subnormal serum concentrations of IgG2. Pediatr Res 27:16–21.

Shackelford PG, Granoff DM, Polmar SH, Scott MG, Goskowicz MC, Madassery JV, Nahm MH. 1990b. Subnormal serum concentrations of IgG2 in children with frequent infections associated with varied patterns of immunologic dysfunction. J Pediatr 116:529–538.

Shemer A, Weiss G, Confino Y, Trau H. 2001. The hyper-IgE syndrome. Two cases and review of the literature. Int J Dermatol 40:622–628.

Sole D, Leser PG, Soares D, Naspitz CK. 1993. Cartilage-hair hypoplasia syndrome: Immunological evaluation of two cases. Rev Paul Med 111:314–319.

Spickett GP, Webster AD, Farrant J. 1990. Cellular abnormalities in common variable immunodeficiency. Immunodefic Rev 2:199–219.

Steimle V, Durand B, Barras E, Zufferey M, Hadam MR, Mach B, Reith W. 1995. A novel DNA-binding regulatory factor is mutated in primary MHC class II deficiency (bare lymphocyte syndrome). Genes Dev 9:1021–1032.

Stiehm ER. 1993. New and old immunodeficiencies. Pediatr Res 33:S2–S7, discussion S-8.

Stray-Pedersen A, Abrahamsen TG, Froland SS. 2000. Primary immunodeficiency diseases in Norway. J Clin Immunol 20:477–485.

Sullivan KE. 2004. The clinical, immunological, and molecular spectrum of chromosome 22q11.2 deletion syndrome and DiGeorge syndrome. Curr Opin Allergy Clin Immunol 4:505–512.

Tangsinmankong N, Bahna SL, Good RA. 2001. The immunologic workup of the child suspected of immunodeficiency. Ann Allergy Asthma Immunol 87:362–369, quiz 70, 423.

Tarzi MD, Jenner M, Hattotuwa K, Faruqi AZ, Diaz GA, Longhurst HJ. 2005. Sporadic case of warts, hypogammaglobulinemia, immunodeficiency, and myelokathexis syndrome. J Allergy Clin Immunol 116:1101–1105.

Tchilian EZ, Wallace DL, Wells RS, Flower DR, Morgan G, Beverley PC. 2001. A deletion in the gene encoding the CD45 antigen in a patient with SCID. J Immunol 166:1308–1313.

Thong YH, Robertson EF, Rischbieth HG, Smith GJ, Binns GF, Cheney K, Pollard AC. 1978. Successful restoration of immunity in the DiGeorge syndrome with fetal thymic epithelial transplant. Arch Dis Child 53:580–584.

Timmers E, de Weers M, Alt FW, Hendriks RW, Schuurman RK. 1991. X-linked agammaglobulinemia. Clin Immunol Immunopathol 61:S83–S93.

Timon M, Arnaiz-Villena A, Rodriguez-Gallego C, Perez-Aciego P, Pacheco A, Regueiro JR. 1993. Selective disbalances of peripheral blood T lymphocyte subsets in human CD3 gamma deficiency. Eur J Immunol 23:1440–1444.

Tsukada S, Saffran DC, Rawlings DJ, Parolini O, Allen RC, Klisak I, Sparkes RS, Kubagawa H, Mohandas T, Quan S, et al. 1993. Deficient expression of a B cell cytoplasmic tyrosine kinase in human X-linked agammaglobulinemia. Cell 72:279–290.

Uribe L, Weinberg KI. 1998. X-linked SCID and other defects of cytokine pathways. Semin Hematol 35:299–309.

Vetrie D, Vorechovsky I, Sideras P, Holland J, Davies A, Flinter F, Hammarstrom L, Kinnon C, Levinsky R, Bobrow M, et al. 1993. The gene involved in X-linked agammaglobulinaemia is a member of the src family of protein–tyrosine kinases. Nature 361:226–233.

ImmunoDeficiency Resource (IDR). In Turku, Finland, Institute of Medical Technology, University of Tampere. Vihinen M. 2004. A comprehensive Web and WAP accessible knowledge base of primary immunodeficiencies.

Vihinen M, Arredondo-Vega FX, Casanova JL, Etzioni A, Giliani S, Hammarstrom L, Hershfield MS, Heyworth PG, Hsu AP, Lahdesmaki A, Lappalainen I, Notarangelo LD, Puck JM, Reith W, Roos D, Schumacher RF, Schwarz K, Vezzoni P, Villa A, Valiaho J, Smith CI. 2001. Primary immunodeficiency mutation databases. Adv Genet 43:103–188.

Villa A, Santagata S, Bozzi F, Imberti L, Notarangelo LD. 1999. Omenn syndrome: a disorder of Rag1 and Rag2 genes. J Clin Immunol 19:87–97.

Villard J, Mach B, Reith W. 1997. MHC class II deficiency: Definition of a new complementation group. Immunobiology 198:264–272.

Villasenor J, Benoist C, Mathis D. 2005. AIRE and APECED: Molecular insights into an autoimmune disease. Immunol Rev 204:156–164.

Walport MJ. 2001a. Complement. First of two parts. N Engl J Med 344:1058–1066.

Walport MJ. 2001b. Complement. Second of two parts. N Engl J Med 344:1140–1144.

Wang Y, Kanegane H, Sanal O, Tezcan I, Ersoy F, Futatani T, Miyawaki T. 2002. Novel Igalpha (CD79a) gene mutation in a Turkish patient with B cell-deficient agammaglobulinemia. Am J Med Genet 108:333–336.

Webster AD, Barnes DE, Arlett CF, Lehmann AR, Lindahl T. 1992. Growth retardation and immunodeficiency in a patient with mutations in the DNA ligase I gene. Lancet 339:1508–1509.

Wildin RS, Smyk-Pearson S, Filipovich AH. 2002. Clinical and molecular features of the immunodysregulation, polyendocrinopathy, enteropathy, X linked (IPEX) syndrome. J Med Genet 39:537–545.

Yabe T, Kawamura S, Sato M, Kashiwase K, Tanaka H, Ishikawa Y, Asao Y, Oyama J, Tsuruta K, Tokunaga K, Tadokoro K, Juji T. 2002. A subject with a novel type I bare lymphocyte syndrome has tapasin deficiency due to deletion of 4 exons by Alu-mediated recombination. Blood 100:1496–1498.

Yel L, Minegishi Y, Coustan-Smith E, Buckley RH, Trubel H, Pachman LM, Kitchingman GR, Campana D, Rohrer J, Conley ME. 1996. Mutations in the mu heavy-chain gene in patients with agammaglobulinemia. N Engl J Med 335:1486–1493.

Zegers BJ, Stoop JW. 1983. Therapy in adenosine deaminase and purine nucleoside phosphorylase deficient patients. Clin Biochem 16:43–47.

Zhang JG, Morgan L, Spickett GP. 1996. L-selectin in patients with common variable immunodeficiency (CVID): A comparative study with normal individuals. Clin Exp Immunol 104:275–279.

Zhou H, Glimcher LH. 1995. Human MHC class II gene transcription directed by the carboxyl terminus of CIITA, one of the defective genes in type II MHC combined immune deficiency. Immunity 2:545–553.

zur Stadt U, Schmidt S, Kasper B, Beutel K, Diler AS, Henter JI, Kabisch H, Schneppenheim R, Nurnberg P, Janka G, Hennies HC. 2005. Linkage of familial hemophagocytic lymphohistiocytosis (FHL) type-4 to chromosome 6q24 and identification of mutations in syntaxin 11. Hum Mol Genet. 14:827–834.

